# Koopman mode decomposition of thermodynamic dissipation in nonlinear Langevin dynamics

**DOI:** 10.1073/pnas.2530617123

**Published:** 2026-06-18

**Authors:** Daiki Sekizawa, Sosuke Ito, Masafumi Oizumi

**Affiliations:** ^a^ Department of General Systems Studies, The University of Tokyo, Meguro-ku, Tokyo 153-8902, Japan; ^b^ Department of Physics, The University of Tokyo, Bunkyo-ku, Tokyo 113-0033, Japan; ^c^ Universal Biology Institute, Graduate School of Science, The University of Tokyo, Bunkyo-ku, Tokyo 113-0033, Japan

**Keywords:** stochastic thermodynamics, Langevin equation, nonlinear phenomena, Koopman mode decomposition

## Abstract

Nonlinear oscillations are commonly observed in complex systems far from equilibrium, such as living organisms. These oscillations are essential for sustaining vital processes, like neuronal firing, circadian rhythms, and heartbeats. In such systems, thermodynamic dissipation is necessary to maintain oscillations against noise. However, due to their nonlinear dynamics, it has been challenging to determine how the characteristics of oscillations, such as frequency, amplitude, and coherent patterns across elements, influence dissipation. To resolve this issue, we employ Koopman mode decomposition, which recasts nonlinear dynamics as a linear evolution in a function space. This linearization allows the dynamics to be decomposed into temporal oscillatory modes coherent across elements, with the Koopman eigenvalues determining their frequencies. Using this method, we decompose thermodynamic dissipation caused by nonconservative forces into contributions from oscillatory modes in overdamped nonlinear Langevin dynamics. We show that the dissipation from each mode is proportional to its frequency squared and its intensity, providing an interpretable, mode-by-mode picture. In the noisy FitzHugh–Nagumo model, we demonstrate the effectiveness of this framework in quantifying the impact of oscillatory modes on dissipation during nonlinear phenomena like coherent resonance and bifurcation. For instance, our analysis of coherent resonance reveals that the greatest dissipation at the optimal noise intensity is supported by a broad spectrum of frequencies, whereas at nonoptimal noise levels, dissipation is dominated by specific frequency modes. Our work offers a general approach to connecting oscillations to dissipation in noisy environments and improves our understanding of diverse oscillation phenomena from a nonequilibrium thermodynamic perspective.

## Significance

Oscillations in nonlinear systems underlie phenomena from chemical waves to neural rhythms. Such oscillations in noisy environments incur thermodynamic dissipation, yet how their frequency, amplitude, and coherence shape this cost remains unclear. Here, using Koopman mode decomposition, which recasts nonlinear dynamics as linear evolution in function space, we show that this cost, measured by the housekeeping entropy production rate, splits into contributions from oscillatory modes, each scaling as the squared frequency times its intensity. The decomposition also implies that, at fixed cost, faster modes face tighter amplitude limits. The framework reveals frequency-resolved structure invisible in the total cost, showing that dissipation can arise from one dominant mode or a broad spectrum in nonlinear phenomena such as bifurcation and coherent resonance.

Oscillations are a pervasive phenomenon in nature. Examples range from the rhythmic beating of the heart (1, 2) and circadian clocks that regulate sleep–wake cycles (3, 4), to neuronal firing patterns (5) and periodic chemical waves in the Belousov–Zhabotinsky reaction (6, 7). These oscillations require physical processes to be far from thermodynamic equilibrium in order to persist (8–27). In the steady state, the extent to which a system deviates from thermodynamic equilibrium is quantified by the entropy production rate (28). The entropy production rate is a nonnegative quantity that measures total irreversibility. It also illustrates the extent to which nonconservative forces violate the detailed balance condition, thereby causing oscillatory behavior in the steady state.

This paper addresses the key question of how the characteristics of oscillations, such as frequency, amplitude, and coherent patterns across elements, determine the entropy production rate. In a previous paper (8), we attempted to answer the question by considering the eigenmode decomposition of the housekeeping entropy production rate (29–33), which is the amount of dissipation caused only by nonconservative forces. However, the analysis was limited to linear forces and could not account for various oscillatory behaviors (34–37), such as limit cycles, bifurcations, in which oscillations suddenly appear or change character, and coherent resonance, in which noise paradoxically stabilizes rhythmic activity. Although several studies have examined the relationship between nonlinear phenomena and thermodynamic dissipation (22, 23, 38–47), the inherent complexity of general nonlinear systems makes it difficult to interpret how their dissipation arises from oscillatory behavior. The direct connection between oscillatory properties and thermodynamic dissipation remains unclear because entropy production is a scalar quantity. For instance, when the entropy production rate changes as a parameter varies, it is challenging to discern whether the change is due to a shift in frequency or amplitude.

To address the aforementioned challenge, we use Koopman mode decomposition (48, 49), a powerful framework for analyzing nonlinear dynamical systems. This method decom- poses nonlinear dynamics into a sum of oscillatory modes. The key concept is that nonlinear dynamics are recast as linear evolution in an infinite-dimensional function space governed by a linear operator known as the Koopman generator. The essential modes that capture this linear evolution can then be identified in a data-driven manner using dynamic mode decomposition (DMD) (50–58). This linearization provides a systematic approach to decomposing a system’s behavior into a set of oscillatory modes, thereby making an otherwise difficult-to-interpret system easier to interpret. This method has also been used to describe noisy nonlinear oscillators (59, 60).

Using Koopman mode decomposition, we establish a general relationship between nonlinear oscillation and the housekeeping entropy production rate in overdamped Langevin systems with general nonlinear forces. Our central result is the decomposition of the housekeeping entropy production rate into a sum of positive contributions from each Koopman mode. Each mode’s contribution is shown to be proportional to the product of its frequency squared and oscillation intensity. This work is a nonlinear extension of our previous study on linear systems (8) and provides an interpretable tool for thermodynamic analysis of noise-induced nonlinear oscillatory phenomena.

Furthermore, to demonstrate the utility of our framework in analyzing nonlinear dynamics, we apply it to the noisy FitzHugh–Nagumo model (61), a canonical model for neural excitability. This enables a mode-by-mode analysis of the entropy production rate in the steady state for oscillations undergoing bifurcation and coherent resonance. Near the bifurcation threshold, our decomposition revealed that an initially broad spectrum of contributions to the entropy production rate experiences intermittent dropouts as the oscillation fades. For coherent resonance, we found that the optimal response to noise was characterized by a broad spectrum of frequency modes, each of which significantly contributed to the total dissipation. These results provide a mode-by-mode picture of how thermodynamic dissipation is structured during complex nonlinear events.

## Background of Stochastic Thermodynamics

Here we explain the setup of our study. We consider the overdamped multidimensional Langevin equation for the dynamics of the state in d-dimensional space, $x_{t}\in R^{d}$, at time t:

[1] $$\begin{array}{c} dx_{t}=D_{t}f_{t}\left(x_{t}\right)dt+\sqrt{2D_{t}}dB_{t}, \end{array}$$

where $dx_{t}$ is the increment of the state; dt is the infinitesimal time interval; $f_{t}\left(x_{t}\right)$ is the force at state $x_{t}$ and time t, and $D_{t}$ is a d×d matrix representing the strength of the noise at time t. We assume that $D_{t}$ is a positive definite matrix, and thus its inverse, $D_{t}^{-1}$, exists. The term $\sqrt{D_{t}}$ represents the unique symmetric positive definite square root of $D_{t}$, which satisfies $D_{t}=\sqrt{D_{t}}{\sqrt{D_{t}}}^{⊤}$, where the superscript ^⊤^ denotes the matrix transpose. The term $dB_{t}$ denotes an increment of a standard d-dimensional Brownian motion, which is a Wiener process satisfying $E[dB_{t}]=0$ and $E\left[dB_{t}dB_{s}^{⊤}\right]=\delta \left(t-s\right)Idt$, where E[·] denotes the expected value and I is the identity matrix.

The Langevin equation can be reformulated using the following Fokker–Planck equation:

[2] $$\frac{\partial p_{t}(x)}{\partial t}=-\nabla \cdot [\nu_{t}(x)p_{t}(x)]$$

[3] $$\begin{array}{cc} \nu_{t}\left(x\right) & =D_{t}\left(f_{t}\left(x\right)-\nabla \ln p_{t}\left(x\right)\right) \end{array}$$

The Fokker–Planck equation is a deterministic equation describing the temporal evolution of the probability distribution $p_{t}\left(x\right)$. The velocity field $\nu_{t}\left(x\right)$ is called the local mean velocity. A system is in a steady state when $p_{t}\left(x\right)$ does not change over time.

The entropy production rate $\sigma_{t}$ is defined as the following $L^{2}$ norm of the local mean velocity $\nu_{t}$ with a metric $D_{t}^{-1}p_{t}\left(x\right)$ (28), i.e.,

[4] $$\begin{array}{c} \sigma_{t}={\left\langle \nu_{t}^{⊤}D_{t}^{-1}\nu_{t}\right\rangle }_{t}, \end{array}$$

where ${\left\langle \cdots \right\rangle }_{t}=\int dx\;p_{t}\left(x\right)\cdots$ denotes the expected value at time t. We assume that the state variables $x_{t}$ have even parity, meaning they are invariant under time reversal and do not include odd-parity variables such as velocity. The nonnegativity of the entropy production rate, $\sigma_{t}\geq 0$, is a statement of the second law of thermodynamics (28).

### Housekeeping Entropy Production Rate.

**Fig. 1.**
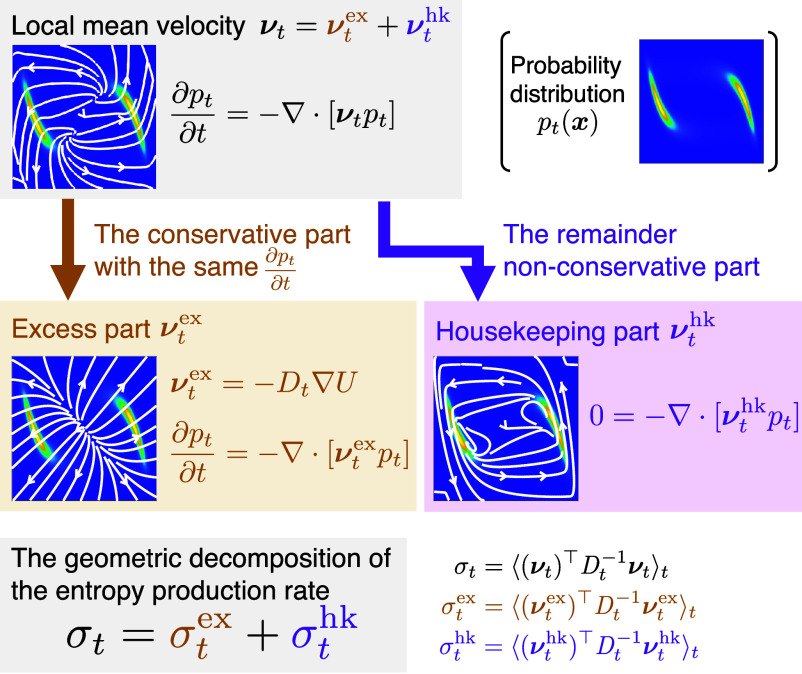
Schematic illustration of the geometric decomposition of the entropy production rate $\sigma_{t}$ into the housekeeping part $\sigma_{t}^{\text{hk}}$ and the excess part $\sigma_{t}^{\text{ex}}$ (32, 62). The excess part $\nu_{t}^{\text{ex}}$ means the velocity field given by the conservative force that provides the same time evolution as the original velocity field $\nu_{t}$. The remainder housekeeping part $\nu_{t}^{\text{hk}}$ corresponds to the nonconservative force and does not contribute to the time evolution of $p_{t}\left(x\right)$. The parts of the entropy production rates associated with the respective parts of the local mean velocities are $\sigma_{t}^{\text{ex}}={\left\langle {\left(\nu_{t}^{\text{ex}}\right)}^{⊤}D_{t}^{-1}\nu_{t}^{\text{ex}}\right\rangle }_{t}$ and $\sigma_{t}^{\text{hk}}={\left\langle {\left(\nu_{t}^{\text{hk}}\right)}^{⊤}D_{t}^{-1}\nu_{t}^{\text{hk}}\right\rangle }_{t}$. We use here the noisy FitzHugh–Nagumo model to describe these schematics.

We consider the housekeeping entropy production rate $\sigma_{t}^{\text{hk}}$ introduced by geometric decomposition (31) (Fig. 1). This housekeeping quantifies dissipation caused by nonconservative forces.

To define the housekeeping entropy production rate, we decompose the local mean velocity $\nu_{t}\left(x\right)$ as $\nu_{t}\left(x\right)=\nu_{t}^{\text{hk}}\left(x\right)+\nu_{t}^{\text{ex}}\left(x\right)$. Here, $\nu_{t}^{\text{ex}}\left(x\right)$ is defined by a potential $U_{t}\left(x\right)$, which satisfies the conditions $\nu_{t}^{\text{ex}}\left(x\right)=-D_{t}\nabla U_{t}\left(x\right)$ and $\partial p\left(x\right)/\partial t=-\nabla \cdot [\nu_{t}^{\text{ex}}\left(x\right)p_{t}\left(x\right)]$. Thus, $\nu_{t}^{\text{ex}}\left(x\right)$ means the velocity field given by the conservative force that provides the same time evolution as the original velocity field $\nu_{t}\left(x\right)$. The remainder $\nu_{t}^{\text{hk}}\left(x\right)$ is the contribution that is not given by the conservative force. Because $\partial p_{t}\left(x\right)/\partial t=-\nabla \cdot \left[\nu_{t}\left(x\right)p_{t}\left(x\right)\right]=-\nabla \cdot \left[\nu_{t}^{\text{ex}}\left(x\right)p_{t}\left(x\right)\right]$ is satisfied, the term $\nu_{t}^{\text{hk}}\left(x\right)$ does not contribute to the time evolution as $-\nabla \cdot [\nu_{t}^{\text{hk}}\left(x\right)p_{t}\left(x\right)]=0$. We note that $\nu_{t}^{\text{hk}}\left(x\right)$ is equivalent to $\nu_{t}\left(x\right)$ if $p_{t}\left(x\right)$ is the steady-state distribution, that is, $\partial p_{t}\left(x\right)/\partial t=0$. However, $\nu_{t}^{\text{hk}}\left(x\right)$ is not generally given by the velocity field in the steady state if $p_{t}\left(x\right)$ is not the steady-state distribution, and it is different from the housekeeping-excess decomposition by Hatano and Sasa (63). The housekeeping entropy production is defined as (31)

[5] $$\begin{array}{c} \sigma_{t}^{\text{hk}}={\left\langle {\left(\nu_{t}^{\text{hk}}\right)}^{⊤}D_{t}^{-1}\nu_{t}^{\text{hk}}\right\rangle }_{t}, \end{array}$$

which means the dissipation caused by the nonconservative contribution $\nu_{t}^{\text{hk}}\left(x\right)$. The difference between the entropy production rate and the housekeeping entropy production rate is given by the excess entropy production rate $\sigma_{t}^{\text{ex}}:=\sigma_{t}-\sigma_{t}^{\text{hk}}={\left\langle {\left(\nu_{t}^{\text{ex}}\right)}^{⊤}D_{t}^{-1}\nu_{t}^{\text{ex}}\right\rangle }_{t}$, and thus the nonnegativity of the excess entropy production rate implies $\sigma_{t}^{\text{hk}}\leq \sigma_{t}$. The equality $\sigma_{t}^{\text{hk}}=\sigma_{t}$ holds in the steady state because $\nu_{t}^{\text{ex}}\left(x\right)$ and $\sigma_{t}^{\text{ex}}$ become zero when $\partial p_{t}\left(x\right)/\partial t=0$. We note that the decomposition $\sigma_{t}=\sigma_{t}^{\text{ex}}+\sigma_{t}^{\text{hk}}$ is called the geometric decomposition because this decomposition is given by a generalization of the Pythagorean theorem (31).

## Koopman Mode Decomposition of the Housekeeping Entropy Production Rate

### Koopman Mode Decomposition of the Virtual Dynamics Driven by $\nu_{t}^{\text{hk}}$.

To prepare for our main result, we will introduce the Koopman mode decomposition to the following virtual dynamical system:

[6] $$\begin{array}{c} dx_{s}=\nu_{t}^{\text{hk}}\left(x_{s}\right)ds, \end{array}$$

where the housekeeping part of the local mean velocity $\nu_{t}^{\text{hk}}\left(x\right)$ is derived from the original process (Eq. **1**) to obtain the housekeeping entropy production rate $\sigma_{t}^{\text{hk}}$. The subscript s stands for the time of the virtual dynamics, whereas t stands for the time of the original Langevin dynamics (Eq. **1**). During the virtual deterministic processes, $\nu_{t}^{\text{hk}}\left(x\right)$ is fixed with respect to changes in s. Let $q_{s}\left(x\right)$ be the probability distribution at time s in this virtual dynamics. The time evolution of $q_{s}\left(x\right)$ is given by $\partial q_{s}\left(x\right)/\partial s=-\nabla \cdot \left[\nu_{t}^{\text{hk}}\left(x\right)q_{s}\left(x\right)\right]$. We note that the probability distribution of the original dynamics $p_{t}\left(x\right)$ becomes the invariant measure of the virtual dynamics. This means that $\partial q_{s}\left(x\right)/\partial s=0$ if $q_{s}\left(x\right)$ is the same as $p_{t}\left(x\right)$.

Introducing the virtual dynamics is not merely a convenience but is necessary for applying the decomposition to the housekeeping local mean velocity field, which includes both the deterministic drift and the diffusion-induced contribution. The oscillations observed in the virtual dynamics therefore incorporate not only the deterministic drift $D_{t}f_{t}\left(x\right)$, but also the diffusion-induced force $-D_{t}\nabla \ln p_{t}\left(x\right)$ from high-density to low-density regions, as defined in Eq. **3**. In this way, the virtual dynamics retain the effective influence of the stochastic term $\sqrt{2D_{t}}\;dB_{t}$, which tends to vanish on average in the original Langevin dynamics. Moreover, this step is not an approximation: Eq. **6** gives an exact deterministic representation of the housekeeping local mean velocity field. As discussed further in *SI Appendix*, *Physical Meaning of Frequencies Extracted in Our Methods* once diffusion-induced transport is included, the oscillatory structure associated with the local mean velocity field is not always fully characterized by the deterministic drift alone. Accordingly, our decomposition captures coherent oscillatory structures shaped jointly by deterministic drift and noise-driven flows.

**Fig. 2.**
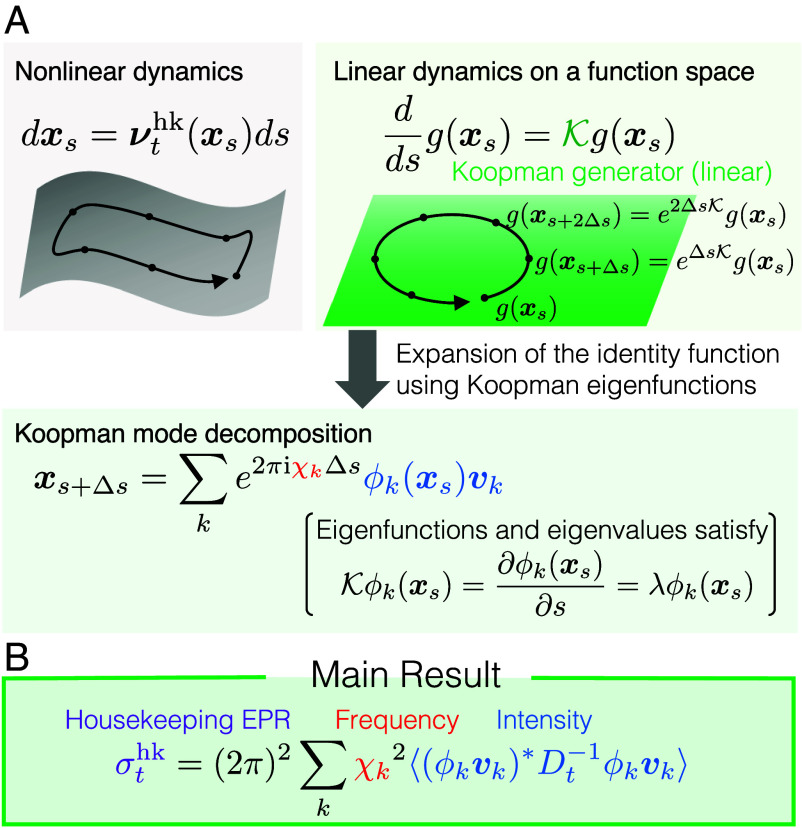
(*A*) Koopman mode decomposition. The virtual dynamics in Eq. **6** are decomposed into a sum of the oscillatory modes using the Koopman mode decomposition. The Koopman generator K transforms a nonlinear dynamical system into the linear dynamics on a function space. Using the eigenvalues ${\left\{\lambda_{k}\right\}}_{k=1}^{r}$ and the eigenfunctions ${\left\{\phi_{k}\right\}}_{k=1}^{r}$ of the Koopman generator K, the time variation of $xx_{s}$ in virtual dynamics can be expressed as a sum of modes. (*B*) Our main result. The housekeeping entropy production rate is decomposed into a sum of contributions from oscillatory modes. Each mode’s contribution is the product of the square of its frequency and its oscillation intensity.

The nonlinear dynamics in Eq. **6** can be represented as linear dynamics by extracting a finite number of modes through Koopman mode decomposition (48, 49) (Fig. 2*A*). As detailed in *SI Appendix*, *Koopman Mode Decomposition of Virtual Dynamics Given by $\nu_{t}^{\text{hk}}$*), the Koopman generator K is defined as a linear operator that maps a function $g:R^{d}\to C$ to another function $Kg:=\nabla g^{⊤}\nu_{t}^{\text{hk}}$. This linear operator satisfies $Kg\left(x_{s}\right)=\left(d/ds\right)g\left(x_{s}\right)$, describing the linear time evolution of the observable $g(x_{s})$. Intuitively, the Koopman generator transforms a nonlinear dynamical system into a linear dynamical system on a function space, which is driven by the linear operator K.

To describe the nonlinear dynamics by exploiting this linearization, we consider an expansion of the identity function Id(x)=x using the eigenvalues ${\left\{\lambda_{k}\right\}}_{k=1}^{r}$ and the eigenfunctions ${\left\{\phi_{k}\right\}}_{k=1}^{r}$ of the Koopman generator K satisfying $K\phi_{k}\left(x\right)=\lambda_{k}\phi_{k}\left(x\right)$. The scalar r is the number of modes. In this study, we assume that the Koopman generator can be accurately approximated by a finite-dimensional linear operator. In the virtual dynamics in Eq. **6**, the Koopman generator K is skew-adjoint with respect to the $L^{2}$ inner product weighted by $p_{t}\left(x\right)$ (*SI Appendix*, *Skew-Adjointness and Diagonalizability of the Koopman Generator*) and is therefore diagonalizable under this finite-dimensional approximation. Then, the identity function is given by $\text{Id}\left(x_{s}\right)=\sum_{k}^{r}\phi_{k}\left(x_{s}\right)v_{k}$ with weights $v_{k}$ called Koopman modes, and the time variation of $x_{s}$ on the virtual dynamics can be written as

[7] $$\begin{array}{cc} x_{s+\Delta s} & =\sum_{k=1}^{r}e^{\lambda_{k}\Delta s}\phi_{k}\left(x_{s}\right)v_{k}=\sum_{k=1}^{r}e^{2\pi \chi_{k}\;\text{i}\Delta s}\phi_{k}\left(x_{s}\right)v_{k}. \end{array}$$

This decomposition is called the Koopman mode decomposition. Here, $\chi_{k}$ is the frequency defined as

[8] $$\begin{array}{c} \chi_{k}=\left|\lambda_{k}/\left(2\pi \;\text{i}\right)\right|, \end{array}$$

where i stands for the imaginary unit. As derived in *SI Appendix*, *Derivation of the Main Result*), the eigenvalue $\lambda_{k}$ is purely imaginary, and hence, $\chi_{k}$ is a real number. This means that the time variation of $x_{s}$ in Eq. **7** can be expressed as the sum of the oscillatory modes.

We also introduce the intensities of the oscillatory modes. When the eigenvalues are not degenerate, the intensity of the k-th oscillatory mode is

[9] $$\begin{array}{c} J_{k}={\left\langle {\left(\phi_{k}v_{k}\right)}^{*}D_{t}^{-1}\left(\phi_{k}v_{k}\right)\right\rangle }_{t}. \end{array}$$

The symbol ^∗^ stands for conjugate transpose. This quantity is the $L^{2}$ norm of the k-th mode $\phi_{k}v_{k}$ in Eq. **7** under the metric $D_{t}^{-1}p_{t}\left(x\right)$, representing the strength of k-th oscillatory mode.

### The Main Result.

Our main result is a decomposition of the housekeeping entropy production rate into independent positive contributions from each oscillatory mode:

[10] $$\begin{array}{cc} \sigma_{t}^{\text{hk}} & =\sum_{k}\sigma_{t}^{\text{hk},(k)}\\ \sigma_{t}^{\text{hk},(k)} & ={\left(2\pi \right)}^{2}\chi_{k}^{2}J_{k}. \end{array}$$

(For the derivation of this decomposition, see *SI Appendix*, *Derivation of the Main Result*). The decomposition means that the contribution of each oscillatory mode to the housekeeping entropy production rate is the product of its frequency squared $\chi_{k}^{2}$ and its intensity $J_{k}$ (Fig. 2*B*). In other words, modes with higher frequencies and greater intensities contribute more to the housekeeping entropy production rate.

When the eigenvalues are degenerate, the decomposition becomes

[11] $$\begin{array}{cc} \sigma_{t}^{\text{hk}} & =\sum_{k^{\prime }}{\left(2\pi \right)}^{2}\chi_{k^{\prime }}^{2}{\langle {\left(\sum_{l\in C_{k^{\prime }}}\phi_{l}v_{l}\right)}^{*}D_{t}^{-1}\left(\sum_{m\in C_{k^{\prime }}}\phi_{m}v_{m}\right)\rangle }_{t}, \end{array}$$

where $C_{k^{\prime }}:=\left\{l|\lambda_{l}=2\pi \chi_{k^{\prime }}\;\text{i}\right\}$ is the set of indices corresponding to the degenerate eigenvalue $2\pi i\chi_{k}$. Here, the index $k^{\prime }$ is defined such that each $\chi_{k^{\prime }}$ is distinct. The summation is taken over only those $k^{\prime }$ for which $\chi_{k^{\prime }}$ has different values, ensuring that no $k^{\prime }$ with the same value of $\chi_{k^{\prime }}$ is selected more than once.

We note that our decomposition in Eq. **10** is an extension of our previous result for linear Langevin systems (8). We can derive the previous result in ref. 8 from Eq. **10** (*SI Appendix*, *Linear Langevin Dynamics*).

We also note that although our method is based on the eigenvalues of the Koopman generator, it differs from previous approaches that rely on the eigenvalues of transition rate matrices in discrete-state Markov processes (9–12, 24, 25, 27). In our framework, we consider a virtual deterministic dynamical system, meaning that all Koopman eigenvalues are purely imaginary. Instead, we would like to point out that this decomposition is similar to the cycle decomposition of housekeeping entropy production rates (62), which is based on Schnakenberg’s network theory (64).

The decomposition also implies a constraint relating the oscillation frequency, oscillation intensity, and thermodynamic cost. Since all contributions in Eq. **10** are nonnegative, each oscillatory mode must satisfy

[12] $$\begin{array}{c} J_{k}\leq \frac{1}{{\left(2\pi \chi_{k}\right)}^{2}}\;\sigma_{t}^{\text{hk}}\leq \frac{1}{{\left(2\pi \chi_{k}\right)}^{2}}\;\sigma_{t}. \end{array}$$

for $\chi_{k}\neq 0$. This inequality shows that, under a finite thermodynamic cost $\sigma_{t}$, the achievable oscillation intensity $J_{k}$ is bounded. In particular, the bound decreases with increasing frequency, indicating that sustaining strong high-frequency oscillations requires a larger entropy production rate.

## Applications to the Noisy FitzHugh–Nagumo Model

**Fig. 3.**
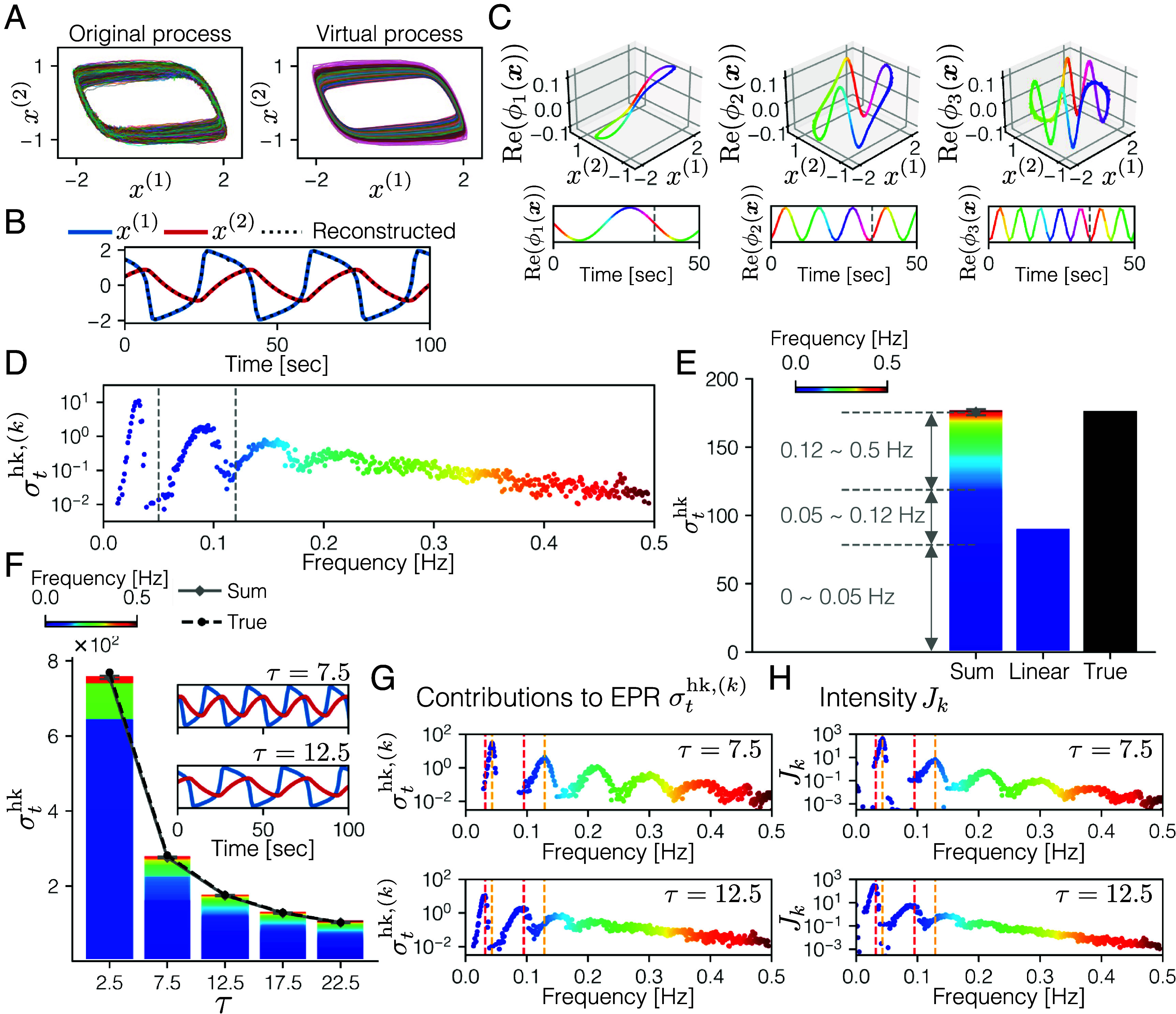
An application example to the noisy FitzHugh–Nagumo model. (*A*) Examples of trajectories that follow the original Langevin process in Eq. **13** and the virtual deterministic process in Eq. **6**. (*B*) An example of a trajectory following the virtual dynamics of the noisy FitzHugh–Nagumo model driven by the housekeeping part of the local mean velocity (Eq. **6**) (colored lines) and the dynamics reconstructed from the Koopman mode decomposition (Eq. **7**) (black dotted line). (*C*) Koopman eigenfunctions $\phi_{k}\left(x\right)$ along an example trajectory. *Top*: The value of $\text{Re}(\phi_{k}\left(x\right))$ at each point along the trajectory. The color represents the time s modulo the period of the slowest oscillation $1/\chi_{1}$, and is consistent with the color used in the *Bottom* panel. *Bottom*: The temporal evolution of $\text{Re}(\phi_{k}\left(x\right))$ along the trajectory. (*D*) The contribution of each oscillatory mode to the housekeeping entropy production rate $\sigma_{t}^{\text{hk},(k)}$. Each dot represents the contribution $\sigma_{t}^{\text{hk},(k)}$. The vertical dashed lines at 0.05 Hz and 0.12 Hz are provided to facilitate comparison with (*E*). *Top*: Result from a single trajectory. *Bottom*: Results from 1,000 trajectories, computed using a moving window over frequency. (*E*) The sum of the contributions from (*D*) almost equals the total value of the housekeeping entropy production rate. *Left*: a stacked bar plot of $\sigma_{t}^{\text{hk},(k)}$. The colors represent the frequencies of the oscillatory modes. The error bars indicate 95% CIs of the sum of the contributions. *Middle*: a stacked bar plot of $\sigma_{t}^{\text{hk},(k)}$ under the assumption of the linear dynamics, calculated using the methods in ref. 8. The colors represent the frequencies of the oscillatory modes. *Right*: the true housekeeping entropy production rate. (*F*) Stacked bar plots showing our decomposition for different values of the time constant τ in Eq. **13**. The stacked bar plots show the sum of the contributions from the oscillatory modes. The colors represent the frequencies of the oscillatory modes. The gray line indicates the sum of the contributions from the oscillatory modes, with error bars representing 95% CIs. The black dashed line shows the true housekeeping entropy production rate. The *Insets* represent examples of the trajectories. (*G*) The contribution of each oscillatory mode to the housekeeping entropy production rate $\sigma_{t}^{\text{hk},(k)}$ for different time constants τ, computed using a moving window over frequency. The vertical dashed lines make it easier to compare peak positions; the red and orange lines respectively indicate the peaks for τ=7.5 and τ=12.5. (*H*) The intensities of the oscillatory modes. The vertical dashed lines also make it easier to compare peak positions; the red and orange lines respectively indicate the peaks for τ=7.5 and τ=12.5.

**Fig. 4.**
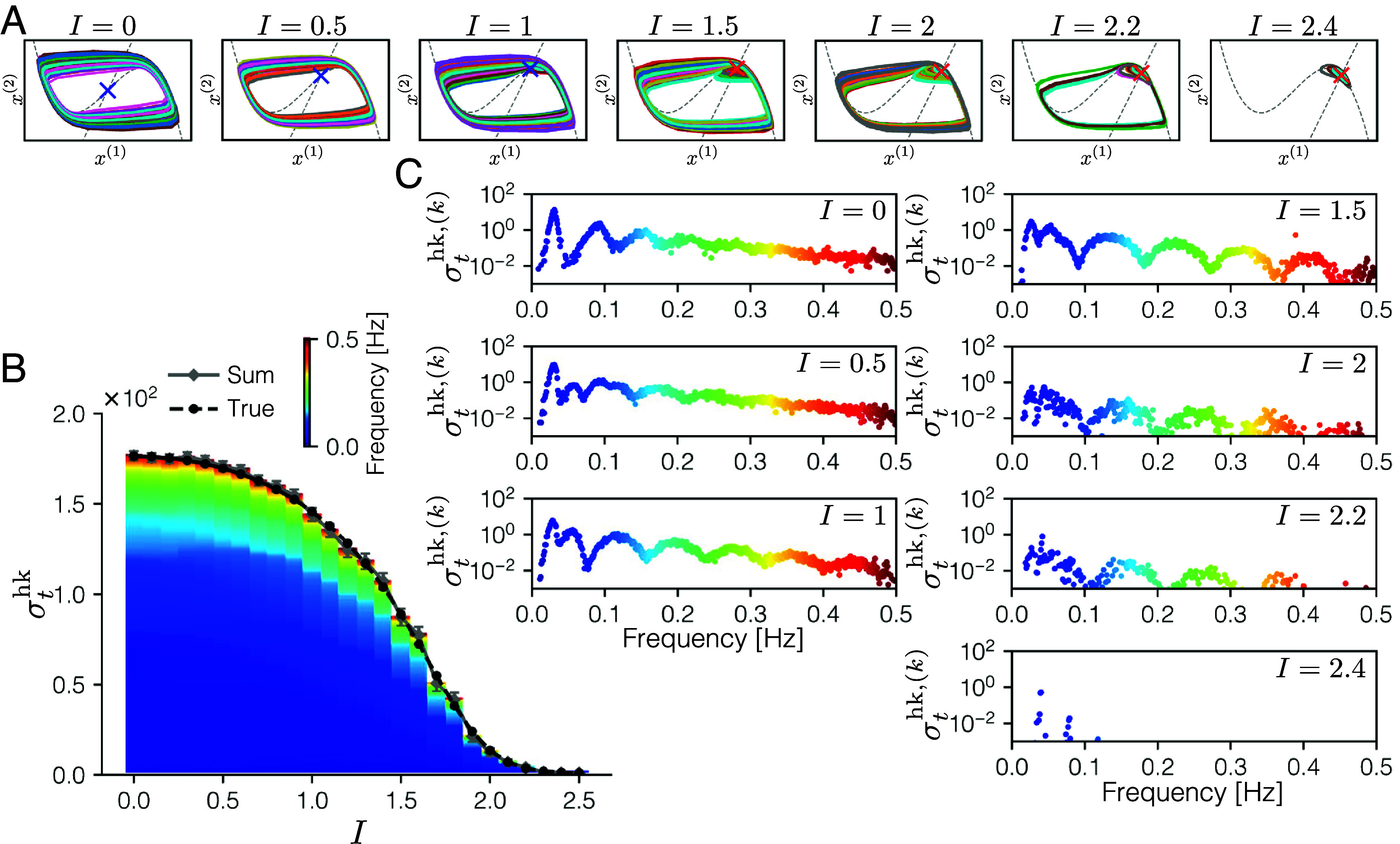
Our decomposition enables us to understand how the entropy production rate depends on the parameter I near the bifurcation point of the noisy FitzHugh–Nagumo model. (*A*) Examples of trajectories of the virtual dynamics in Eq. **6** for the noisy FitzHugh–Nagumo model for different values of I. The black dashed line represents the nullclines of the noisy FitzHugh–Nagumo model, which were calculated from the original Langevin dynamics in Eq. **13** by ignoring the noise term. The blue and red crosses represent the unstable and stable fixed points, respectively. For I<1.5, the trajectory forms a large loop around the unstable fixed point. However, near I=1.5, a stable fixed point emerges, and the trajectory transitions to a smaller loop around this stable point. (*B*) The parameter-dependent behavior of the housekeeping entropy production rate and its decomposition. The stacked bar plot shows the sum of the contributions from the oscillatory modes. The colors represent the frequencies of the oscillatory modes. The gray line indicates the sum of the contributions from the oscillatory modes, with error bars representing 95% CIs. The black dashed line shows the true housekeeping entropy production rate. As the trajectory transitions from the large loop to the small loop, the housekeeping entropy production rate $\sigma_{t}^{\text{hk}}$ significantly decreases. (*C*) The contribution of each oscillatory mode to the housekeeping entropy production rate $\sigma_{t}^{\text{hk},(k)}$ for different input values of I. For I<2.4, a variety of frequencies contribute to the housekeeping entropy production rate $\sigma_{t}^{\text{hk}}$. As I approaches 2.4, the contributions from frequencies undergo intermittent dropout. At I=2.4, almost a single frequency predominantly contributes to the housekeeping entropy production rate $\sigma_{t}^{\text{hk}}$.

**Fig. 5.**
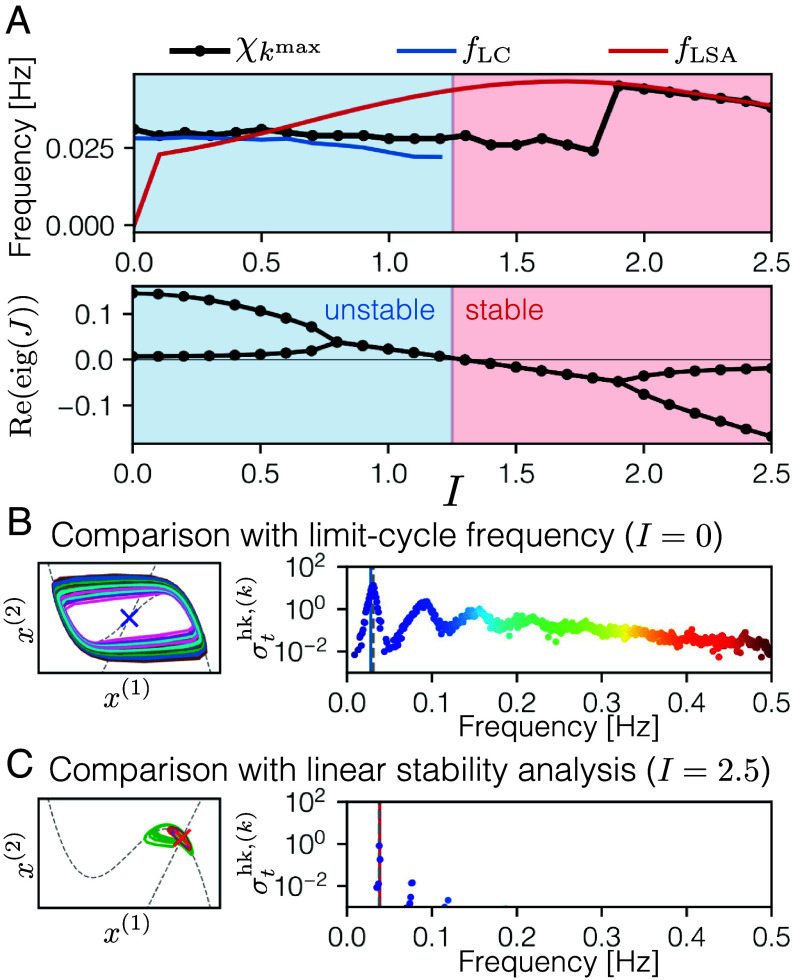
Comparison between the oscillatory frequency extracted by our decomposition and i) the limit cycle frequency $f_{\text{LC}}$ and ii) the frequency $f_{\text{LSA}}$ predicted by linear stability analysis of the local mean velocity field $\nu_{t}^{\text{hk}}$. (*A*) *Top*: Frequency $\chi_{k^{\text{max}}}\left(>0\right)$ contributing most strongly to the housekeeping entropy production rate (black dots), plotted as a function of the input current I. The blue curve indicates the limit cycle frequency $f_{\text{LC}}$ when it exists. The red curve shows the frequency $f_{\text{LSA}}$ obtained from the imaginary part of the eigenvalues of the Jacobian of $\nu_{t}^{\text{hk}}$ evaluated at its fixed point satisfying $\nu_{t}^{\text{hk}}=0$. *Bottom*: Real parts of the two eigenvalues of the Jacobian of the deterministic drift, $J=\partial \left(D_{t}f_{t}\right){/\partial x|}_{x=x_{\mathrm{dfix}}}$, at the deterministic fixed point $xx_{\mathrm{dfix}}$ (i.e., $D_{t}f_{t}\left(x_{\mathrm{dfix}}\right)=0$), indicating unstable (blue shaded) and stable (red shaded) regimes. (*B* and *C*) Examples in the limit cycle regime (I=0) and the stable fixed point regime (I=2.5), respectively. *Left*: Examples of trajectories of the virtual dynamics. The blue and red crosses indicate the unstable and stable fixed points, respectively. *Right*: Contributions of oscillatory modes to the housekeeping entropy production rate. The gray vertical line indicates $\chi_{k^{\text{max}}}$, while the blue and red vertical lines indicate $f_{\text{LC}}$ and $f_{\text{LSA}}$, respectively.

**Fig. 6.**
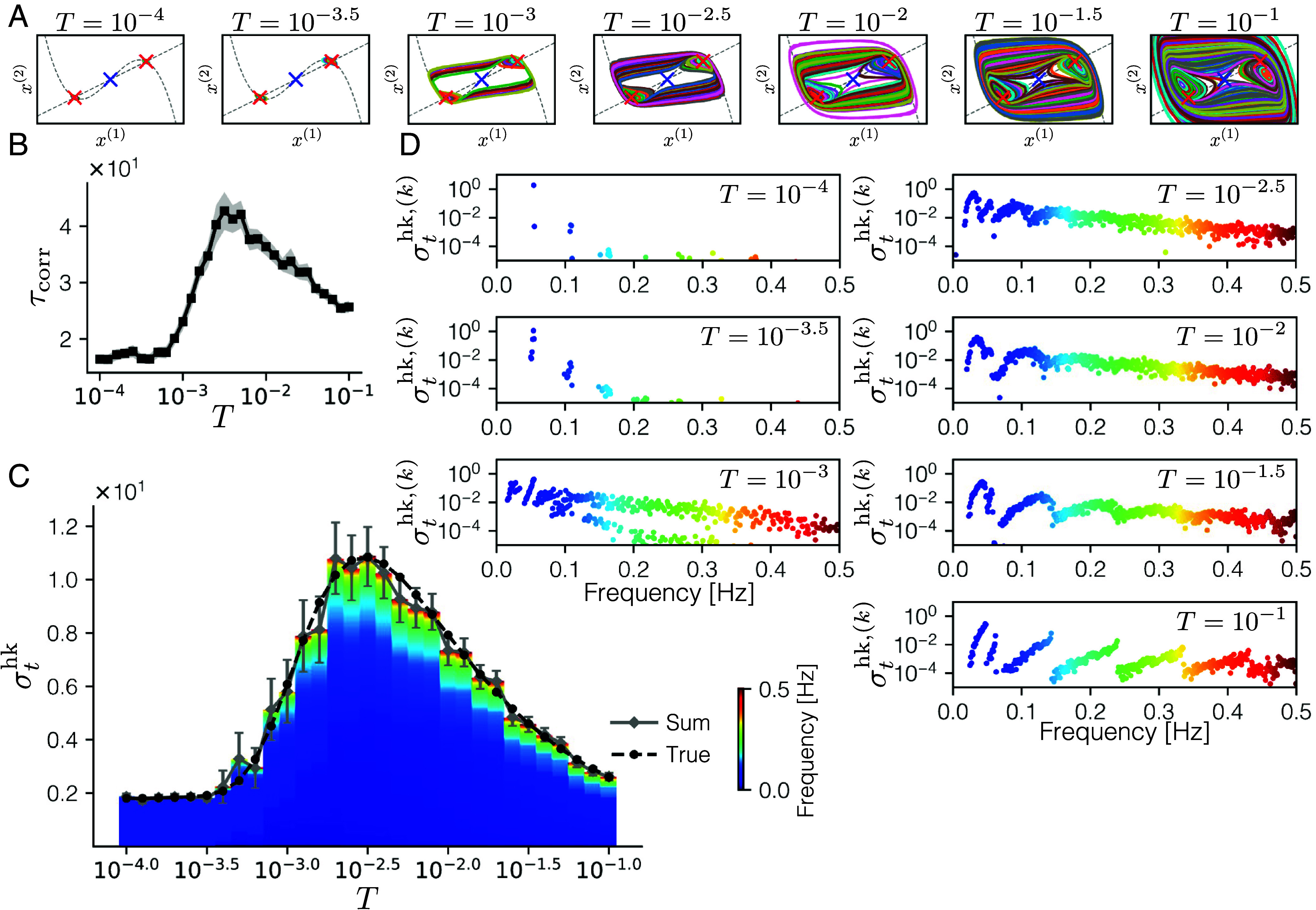
Our decomposition enables us to determine how the entropy production rate depends on the parameter T in the context of coherent resonance in the noisy FitzHugh–Nagumo model. (*A*) Examples of the trajectories of the virtual dynamics in Eq. **6** for the noisy FitzHugh–Nagumo model for different values of T. The black dashed line represents the nullclines of the noisy FitzHugh–Nagumo model, which were calculated from the original Langevin dynamics in Eq. **13** by ignoring the noise term. The blue and red crosses represent the unstable and stable fixed points, respectively. For $T\leq 10^{-3.5}$, the trajectories are trapped in the small loop near the two fixed points. However, for $10^{-3.5}<T$, the trajectories transition between the two stable points, forming large loops in phase space. (*B*) The correlation times $\tau_{\mathrm{corr}}$, required to detect coherent resonance. The shaded areas represent 95% CIs. (*C*) The parameter-dependent behavior of the housekeeping entropy production rate and its decomposition. The stacked bar plot shows the sum of the contributions from the oscillatory modes. The colors represent the frequencies of the oscillatory modes. The gray line indicates the sum of the contributions from the oscillatory modes, with error bars representing 95% CIs. The black dashed line shows the true housekeeping entropy production rate. This curve exhibits an inverted-U shape, which is characteristic of coherent resonance. (*D*) The contribution of each oscillatory mode to the housekeeping entropy production rate $\sigma_{t}^{\text{hk},(k)}$ for different T. When T is small, only one frequency mode significantly contributes to the housekeeping entropy production rate $\sigma_{t}^{\text{hk}}$. As T increases, a broader range of frequency modes begins to contribute. As the total entropy production rate begins to decrease at higher noise intensities, contributions from oscillatory modes gradually decrease.

We demonstrate our decomposition using the FitzHugh–Nagumo model (61) in the presence of noise. This analysis has two main objectives: i) to facilitate an intuitive understanding of our decomposition in a nonlinear setting, and ii) to demonstrate its utility in investigating how nonlinear oscillatory phenomena generate the housekeeping entropy production rate. To achieve the first objective, Fig. 3 illustrates how our decomposition represents the housekeeping entropy production rate through oscillatory modes. For the second objective, in Figs. 4–6, we apply the decomposition to bifurcations and coherent resonance. Throughout, we analyze the steady state, so that the housekeeping entropy production rate coincides with the steady-state entropy production rate. To estimate the Koopman modes and eigenfunctions used in our decomposition, we employed dynamic mode decomposition (DMD) (54–58). The analysis methods are detailed in *Materials and Methods*. We note that the extension to non-steady-state settings remains feasible in a low-dimensional relaxation process; a representative example and the corresponding numerical procedures are given in *SI Appendix*, *Application of Our Decomposition to Non-Steady-State Dynamics* and *Supplementary Methods*.

The noisy FitzHugh–Nagumo model is given by the following Langevin equation:

[13] $$d\left(\begin{array}{c} x_{t}^{\left(1\right)}\\ x_{t}^{\left(2\right)} \end{array}\right)=\left(\begin{array}{c} (x_{t}^{\left(1\right)}-\frac{{\left(x_{t}^{\left(1\right)}\right)}^{3}}{3}-x_{t}^{\left(2\right)}+I\\ \frac{1}{\tau }(x_{t}^{\left(1\right)}+a-bx_{t}^{\left(2\right)}) \end{array}\right)dt+\sqrt{2T}dB_{t}$$

The superscripts ^(*i*)^ for i∈{1,2} represent indices of the dimensions of the state vector $x_{t}={\left(x_{t}^{\left(1\right)},x_{t}^{\left(2\right)}\right)}^{⊤}$. The noisy FitzHugh–Nagumo model is a neuronal model (61) with a membrane potential $x_{t}^{\left(1\right)}\in R$ and a recovery variable $x_{t}^{\left(2\right)}\in R$ of a neuron. The parameters a∈R and b∈R reflect properties of the neuron. I∈R is an input to the neuron. $\tau \in R_{>0}$ is the time constant of the recovery variable. $T\in R_{>0}$ is the intensity of the noise. For Fig. 3, we chose the following values: $a=0,b=0.5,I=0,T=10^{-3}$. The parameter τ=12.5 is used for Fig. 3*A*–*E*, and the parameter τ varies for Fig. 3*F*–*H*. With these parameter values, the dynamics exhibit an oscillatory pattern (Fig. 3*A*, *Left*).

### Demonstration of Our Decomposition.

In this section, we illustrate our decomposition. To prepare for this, we numerically calculated the housekeeping part of the local mean velocity $\nu_{t}^{\text{hk}}\left(x\right)$ and simulated time-series data that follow the virtual dynamics driven by $\nu_{t}^{\text{hk}}\left(x\right)$, as defined in Eq. **6**, which mimics the original Langevin dynamics in Eq. **13** (see Fig. 3*A*, *Right* and *B*, colored lines).

We applied the Koopman mode decomposition to each simulated trajectory to characterize how the virtual dynamics can be expressed in terms of oscillatory modes. From this analysis, we obtained the eigenvalues ${\left\{\lambda_{k}\right\}}_{k=1}^{r}$, eigenfunctions ${\left\{\phi_{k}\right\}}_{k=1}^{r}$, and Koopman modes ${\left\{v_{k}\right\}}_{k=1}^{r}$ associated with the virtual dynamics. In the example trajectory shown in Fig. 3*B*, the trajectory was well reconstructed using 27 modes (black dashed line) (*Materials and Methods*). The real parts of the eigenfunctions, Re(ϕk(x)), exhibit oscillatory variations as x evolves along the trajectory with nonuniform speed, returning to their original values after one cycle (Fig. 3*C*, *Top*). When plotted against time, Re(ϕk(x)) displays sinusoidal waveforms (Fig. 3*C*, *Bottom*). These results indicate that the dynamics of the virtual system can be interpreted as a superposition of oscillatory modes (Eq. **7**), from which the time series can be faithfully reconstructed (see Fig. 3*B*, black dotted line). In the calculation of our decomposition, the expectations are evaluated by Monte Carlo sampling over many trajectories of this virtual dynamics. Because the true underlying Langevin dynamics is known in the present setting, any discrepancy caused by an insufficient number of sampled trajectories can in practice be checked and reduced by increasing the sample size until convergence is reached while monitoring the CIs. See *SI Appendix*, *Finite Sampling Effects in Low Noise Metastable Systems* for details.

From our decomposition in Eq. **10**, we obtain the contributions of the oscillatory modes to the housekeeping entropy production rate $\sigma_{t}^{\text{hk},(k)}$ (Fig. 3 *D* and *E*). We observe large contributions from frequencies around 0.03 Hz, 0.08 Hz, and so on (Fig. 3*D*). The sum of these contributions recovers the total housekeeping entropy production rate $\sigma_{t}^{\text{hk}}$ (Fig. 3*E*, *Left*). This result is consistent with the true housekeeping entropy production rate calculated numerically using the methods in ref. 65 (Fig. 3*E*, *Right*), indicating the validity of our decomposition.

In contrast, when applying the method in ref. 8, which assumes linear Langevin dynamics, the sum of the contributions of modes does not recover the true housekeeping entropy production rate $\sigma_{t}^{\text{hk}}$ (Fig. 3*E*, *Middle*). This result demonstrates the necessity of handling nonlinear dynamics in our decomposition to accurately capture the contributions of oscillatory modes in nonlinear systems.

Next, we analyzed how the decomposition varies with the time constant τ, to demonstrate how our decomposition reveals frequency-dependent features of thermodynamic dissipation that are not accessible from the total entropy production rate alone. As τ increases, the virtual dynamics exhibit oscillations with lower frequencies (Fig. 3*F*, *Insets*), and the total housekeeping entropy production rate $\sigma_{t}^{\text{hk}}$ without decomposition decreases correspondingly (see Fig. 3*F*, black dashed line). Our decomposition allows us to interpret this decrease in terms of frequency-resolved contributions of oscillatory modes. As τ increases, modes with smaller frequencies become dominant contributors to $\sigma_{t}^{\text{hk}}$ (Fig. 3 *F* and *G*), and the overall dissipation decreases because each mode’s contribution $\sigma_{t}^{\text{hk},(k)}$ scales with the square of its frequency (Eq. **10**). This correspondence between frequency and dissipation is consistent with the theoretical structure of our decomposition, which attributes higher energetic costs to faster oscillations. Despite the frequency shift between τ=7.5 and τ=12.5, the peak intensities remain nearly constant (Fig. 3*H*), indicating that the reduction in $\sigma_{t}^{\text{hk}}$ primarily arises from lower oscillation frequencies rather than changes in their amplitudes.

### Application of Our Decomposition Across Bifurcation Regimes.

Building on the demonstration in the previous sections, we next apply our decomposition to investigate how the housekeeping entropy production rate arises near the bifurcation points of the noisy FitzHugh–Nagumo model, thereby demonstrating its utility for analyzing thermodynamic dissipation in nonlinear phenomena. The FitzHugh–Nagumo model is known to exhibit bifurcations in which the qualitative behavior of the system changes depending on parameters such as I and b (37, 61). Fig. 4*A* shows the trajectories of the virtual dynamics in Eq. **6** for various values of I. When I is small, the trajectory forms a large loop, whereas, as I increases, it transitions to a smaller loop. In the noise-free limit, this transition corresponds to a Hopf bifurcation (66), beyond which the small loop disappears. However, in the noisy FitzHugh–Nagumo model, the small loop persists even after the bifurcation due to the presence of weak noise.

Prior to analyzing the housekeeping entropy production rate, we first review how bifurcations emerge in the noisy FitzHugh–Nagumo model by examining the fixed points (37). Fig. 4*A* shows the fixed points calculated by ignoring the noise term in the original Langevin system (Eq. **13**). Unstable fixed points are indicated by blue crosses, and stable fixed points are indicated by red crosses. As the parameter I increases, an unstable fixed point becomes stable around I=1.5 (Fig. 4*A*). Trajectories that initially form large loops with small I values begin shifting toward smaller loops as they approach the Hopf bifurcation. As I increases further, the small loops gradually become dominant. This gradual transition, rather than an abrupt change, reflects the influence of diffusion due to the noise term.

Before applying our decomposition, we examined the total housekeeping entropy production rate and found that it exhibits a gradual decrease near the bifurcation points (Fig. 4*B*). As the input parameter I increases toward I=1.5, where an unstable fixed point becomes stable, the total housekeeping entropy production rate decreases sharply, corresponding to the transition from large loops to small loops (Fig. 4*A*). With further increases in I, the total housekeeping entropy production rate approaches zero around I=2.4, reflecting the dominance of small-loop trajectories around stable fixed points. Although this decrease in the total housekeeping entropy production rate captures the overall effect of the bifurcation, it offers no insight into how individual oscillatory modes contribute to this change.

Qualitative changes in the underlying contributions to the housekeeping entropy production rate from oscillatory modes are revealed only through our decomposition. When the input parameter I is small (around I≃0), oscillatory modes spanning a broad range of frequencies significantly contribute to the entropy production rate (Fig. 4*C*). However, as I increases around the bifurcation point I≃1.5, these contributions exhibit intermittent dropouts in specific frequency bands. This behavior is consistent with the observation that some trajectories become trapped in small loops around stable fixed points. These small loops are associated with low-intensity oscillations that negligibly contribute to the entropy production rate in our decomposition (Eq. **10**). At around I=2.4, one frequency dominates, exceeding all others by nearly two orders of magnitude. This occurs because, in the weak-noise limit, the system behaves like a two-dimensional linear dynamical system around a stable fixed point. In such cases, only one pair of complex conjugate eigenvalues remains, resulting in a single dominant oscillatory mode. Together, these results demonstrate that our decomposition provides insights unavailable from the total entropy production rate alone, offering a quantitative means to understand how bifurcations in nonlinear systems shape their thermodynamic dissipation.

### Physical Interpretation of the Oscillatory Frequencies Extracted by Our Decomposition.

Having analyzed how the entropy production rate is distributed among oscillatory modes across the bifurcation, we next interpret the dominant frequencies extracted by our decomposition by comparing them with two characteristic frequencies in this system: i) the limit cycle frequency $f_{\text{LC}}$ and ii) the frequency $f_{\text{LSA}}$ predicted by linear stability analysis (Fig. 5). The relation to iii) the frequencies associated with the stochastic generator spectrum is also discussed in *SI Appendix*, *Physical Meaning of Frequencies Extracted in Our Methods*.

For this analysis, the key theoretical point is that the oscillatory frequencies extracted by our decomposition are naturally associated with the local mean velocity field $\nu_{t}^{\text{hk}}$, which is determined by both the deterministic drift $D_{t}f_{t}\left(x\right)$ and the diffusion-induced contribution $-D_{t}\nabla \ln p_{t}\left(x\right)$. A more detailed discussion of these two contributions is given in *SI Appendix*, *Physical Meaning of Frequencies Extracted in Our Methods*. Here, we illustrate this interpretation through the representative noisy FitzHugh–Nagumo example in Fig. 5, showing how the dominant frequency in our decomposition is related to the limit-cycle frequency and to the frequency predicted by linear stability analysis of $\nu_{t}^{\text{hk}}$.

To compare the dominant frequency extracted by our decomposition with these reference frequencies, we introduce the quantities used in Fig. 5. Here, $f_{\text{LC}}$ denotes the limit-cycle frequency, defined as the reciprocal of the mean time required for the phase of the Langevin dynamics to advance by one cycle; its detailed definition and numerical evaluation are described in *SI Appendix*, *Supplementary Methods*. We next define the frequency predicted by linear stability analysis and the dominant frequency extracted by our decomposition. Let $x_{\text{fix}}$ be the fixed point satisfying $\nu_{t}^{\text{hk}}\left(x_{\text{fix}}\right)=0$, and let $\lambda_{\text{LSA}}$ denote the eigenvalue of the Jacobian $\partial \nu_{t}^{\text{hk}}{/\partial x|}_{x=x_{\text{fix}}}.$ In the present two-dimensional noisy FitzHugh–Nagumo setting, the eigenvalues form either a complex conjugate pair or a pair of real eigenvalues, so $\lambda_{\text{LSA}}$ is uniquely defined whenever the eigenvalues are complex. We then define $f_{\text{LSA}}=\left|\;\text{Im}\left(\lambda_{\text{LSA}}\right)\right|/\left(2\pi \right)$, which represents the oscillation frequency predicted by linear stability analysis of $\nu_{t}^{\text{hk}}$ around its fixed point. We also introduce $k^{\text{max}}=\underset{k}{\arg\;\max}\;\sigma_{t}^{\text{hk},(k)}$, where $\chi_{k^{\text{max}}}$ is the frequency defined by (Eq. **8**) with $k=k^{\text{max}}$, namely, the frequency contributing most strongly to the housekeeping entropy production rate in our decomposition. Here, $k^{\text{max}}$ does not depend on t because we analyze the steady state. This figure uses the same parameters as Fig. 4.

With this analysis, we found that in the noisy FitzHugh–Nagumo system studied here, the dominant frequency $\chi_{k^{\text{max}}}$ extracted by our decomposition roughly coincides with the limit-cycle frequency $f_{\text{LC}}$ in the unstable regime and with the frequency $f_{\text{LSA}}$ predicted by linear stability analysis in the stable regime. In Fig. 5*A*, *Bottom*, the real parts of the eigenvalues of the Jacobian $J=\partial \left(D_{t}f_{t}\right){/\partial x|}_{x=x_{\text{dfix}}}$ classify the deterministic fixed point $x_{\text{dfix}}$ (i.e., $D_{t}f_{t}\left(x_{\mathrm{dfix}}\right)=0$) into an unstable regime at small I (blue shaded region) and a stable regime at large I (red shaded region). In the present system, these correspond to a limit cycle regime and a regime in which the deterministic dynamics remain near a stable fixed point, respectively. Fig. 5*A*, *Top* shows, for each value of the input current I, the frequency $\chi_{k^{\text{max}}}$ together with the limit cycle frequency $f_{\text{LC}}$ when it exists (blue curve) and the frequency $f_{\text{LSA}}$ predicted from the fixed-point analysis of $\nu_{t}^{\text{hk}}$ (red curve). As shown there, $\chi_{k^{\text{max}}}$ roughly coincides with $f_{\text{LC}}$ throughout the unstable regime, whereas it roughly coincides with $f_{\text{LSA}}$ throughout the stable regime. This correspondence is illustrated by representative examples: At I=0, where the dynamics exhibit limit-cycle-like behavior, $\chi_{k^{\text{max}}}$ agrees with $f_{\text{LC}}$ (Fig. 5*B*), whereas at I=2.5, where the dynamics remain near a stable fixed point, $\chi_{k^{\text{max}}}$ agrees with $f_{\text{LSA}}$ (Fig. 5*C*).

Taken together, these results provide a physical interpretation of the oscillatory frequencies identified by our decomposition. In the present example, these frequencies track the relevant oscillatory motion in each regime: the limit cycle frequency in the unstable regime and the fixed-point oscillation frequency of $\nu_{t}^{\text{hk}}$ in the stable regime.

### Application of Our Decomposition to Coherent Resonance.

Next, we applied our decomposition to investigate how oscillatory modes shape the housekeeping entropy production rate in a coherent-resonance regime of the noisy FitzHugh–Nagumo model. In this regime, noise induces temporally regular oscillatory behavior even in the absence of periodic external input (67, 68). This phenomenon is closely related to stochastic resonance as a form of noise-induced regularity.

The virtual dynamics driven by the housekeeping local mean velocity $\nu_{t}^{\text{hk}}$ in Eq. **6** of the noisy FitzHugh–Nagumo model with parameters a=0, b=2, I=0, and τ=12.5 exhibit coherent resonance as a function of the noise intensity T (Fig. 6*A*). With this parameter setting, the original Langevin system (Eq. **13**) possesses two stable fixed points (see Fig. 6*A*, red crosses). When the noise intensity T is small, the trajectories of the virtual dynamics remain confined near one of the stable points and rarely transition to the other. As T increases, the system begins to transition between the two stable points, forming large loops in phase space due to noise-induced switching. This noise-induced regularization of the dynamics can also be quantitatively characterized by the correlation time $\tau_{\text{corr}}$, which measures the degree of temporal coherence in dynamical systems, as defined in *SI Appendix*, Eq. **S54**. As shown in Fig. 6*B*, $\tau_{\text{corr}}$ forms an inverted U-shaped curve with respect to the noise intensity T, indicating that both weak and strong noise lead to disordered dynamics, while an intermediate noise level produces the highest temporal coherence.

In line with the changes in trajectory structure described above, the total housekeeping entropy production rate, without applying our decomposition, also exhibits an inverted-U-shaped dependence on the noise intensity T, peaking around $T≃10^{-2.5}$ (Fig. 6*C*). It remains small when the system stays near one of the stable fixed points ($T≃10^{-4}$), increases as transitions between the two basins emerge ($T≃10^{-2.5}$), and decreases again under strong noise ($T≃10^{-1}$), where the dynamics approach near-equilibrium behavior. Although this overall trend reflects the characteristic signature of coherent resonance, it does not reveal how individual oscillatory modes contribute to the underlying thermodynamic dissipation.

The underlying contributions of oscillatory modes to the housekeeping entropy production rate can again be revealed only through our decomposition; the observed peak in the housekeeping entropy production rate originates from broad frequency contributions (Fig. 6*D*). At low noise intensities ($T≃10^{-4}$), the entropy production rate is dominated by a single low-frequency mode corresponding to localized motion near a stable fixed point. As the noise level increases ($T≃10^{-2.5}$), additional frequency modes appear, showing that thermodynamic dissipation is distributed across a broader range of oscillatory components. For even higher noise levels ($T≃10^{-1}$), several frequency components selectively vanish, producing a harmonic-like, stepwise pattern in the modal spectrum. These results highlight how our decomposition exposes frequency-resolved features of coherent resonance that cannot be inferred from the total entropy production rate alone.

## Summary and Discussion

This study improves our understanding of nonlinear oscillatory phenomena by providing a thermodynamic framework that quantifies the influence of oscillatory frequency and amplitude on the housekeeping entropy production rate (or the entropy production rate in the steady state) within noise-induced nonlinear systems. Using the Koopman mode decomposition, we analyze the dissipation of oscillations in general nonlinear dynamics with noise by decomposing the housekeeping entropy production rate $\sigma_{t}^{\text{hk}}$ into contributions from oscillatory modes. We applied this framework to the noisy FitzHugh–Nagumo model, using distinct parameter regimes corresponding to bifurcation and coherent resonance, respectively. This revealed how Koopman modes of different frequencies contribute to the entropy production rate in the steady state of these nonlinear phenomena. Thus, the framework provides a clear explanation of dissipation in noise-induced nonlinear systems.

The present study provides a theoretical perspective for understanding how functional oscillations are organized and maintained under finite thermodynamic cost. In living and synthetic systems, oscillatory dynamics are essential for realizing diverse functions (1–7) while operating within thermodynamic constraints (8–22, 24–27). The proposed framework reveals how individual oscillatory modes contribute to the overall thermodynamic dissipation, providing a structured basis for linking oscillatory dynamics with thermodynamic cost. This perspective offers a means to examine how oscillatory systems achieve and regulate function under limited thermodynamic resources and may, in the future, help clarify how efficiency and design principles emerge across biological, physical, and engineered systems.

We clarify the scope and assumptions underlying our framework. In Eq. **7**, our framework is based on the diagonalizability of the Koopman generator under a finite-dimensional numerical approximation. Although the Koopman generator is not generally diagonalizable (69), this treatment is justified when the number of Koopman modes is finite, because K is skew-adjoint in the virtual dynamics in Eq. **6** (*SI Appendix*, *Skew-Adjointness and Diagonalizability of the Koopman Generator*) and thus diagonalizable. However, this approach may not be applicable if there are infinitely many Koopman modes. For example, chaotic systems are known to possess continuous spectra in their Koopman generators (55, 56), which may not be fully captured by a finite number of eigenvalues. Intuitively, this implies that chaotic systems cannot be expressed as a superposition of a finite number of oscillatory modes. It is unclear how valid Eq. **7** is when such a system is forced into a finite-dimensional approximation. If our framework is ineffective in systems with continuous spectra, this suggests that the virtual dynamics cannot be accurately represented by a finite set of oscillatory modes. Nevertheless, dynamic mode decomposition (DMD), which is a data-driven method to estimate the Koopman modes and eigenfunctions from time-series data, has been generalized to handle systems with continuous spectra (51, 52, 56, 70). Therefore, incorporating these approaches could enable us to extend our decomposition to systems for which a finite-dimensional approximation of the Koopman generator is not exactly valid.

Although our analysis focuses on simplified dynamical models, Eq. **12** may provide insight into energetic constraints underlying neural oscillations. In the FitzHugh–Nagumo model, oscillatory dynamics are often interpreted as simplified representations of neuronal activity. The bound in Eq. **12** suggests that, if oscillatory modes correspond to neural rhythms, higher-frequency oscillations would require larger thermodynamic dissipation to achieve the same oscillation intensity. While the FitzHugh–Nagumo model is not intended to quantitatively describe metabolic energy consumption in neurons, this result highlights a potential thermodynamic constraint linking oscillation frequency, oscillation strength, and dissipative cost. Such constraints may offer a conceptual perspective on how energetic limitations could influence the observable frequencies and amplitudes of neural oscillations.

A potential difficulty of the present approach is that estimation becomes more difficult in higher-dimensional chaotic systems. One reason is that such systems can involve continuous or broadened spectral components, so a description based on only a small number of oscillatory modes may no longer be sufficient. In addition, higher-dimensional chaotic systems can make it more difficult to simulate long trajectories of the virtual dynamics with sufficient numerical accuracy. They can also make it harder to determine a state-space discretization that is fine enough to evaluate the underlying entropy production rate reliably. For these reasons, the required number of modes and the numerical difficulty are expected to depend on the structure of the specific system, and we leave this issue for future work.

A promising direction for future research is to apply our decomposition framework to real data from noisy oscillatory systems. However, doing so would present practical challenges. Such an application would require addressing two issues simultaneously: i) performing the Koopman mode decomposition of the housekeeping entropy production rate and ii) carrying out the geometric decomposition of the velocity field into housekeeping and excess parts. In this setting, finite-sample effects may further complicate both tasks, especially in low-noise metastable systems where rare transitions and low-probability but highly dissipative regions may be undersampled. Nevertheless, when this issue is not severe, both i) and ii) may still be tractable with existing methods. Regarding i), methods have been actively developed to identify Koopman eigenvalues, eigenfunctions, and modes from time series data using data-driven approaches (50–58). These methods may provide a foundation for implementing the Koopman analysis in practice. Regarding ii), recent advances have proposed methods for estimating the local mean velocity and its decomposition into housekeeping and excess parts from optimization problems based on thermodynamic uncertainty relations and optimal transport theory (31–33, 71–73). Thus, the housekeeping part of the velocity field may be obtained numerically from time series data via an optimization problem. Furthermore, incorporating the concept of inferring the lower bound on the entropy production rate under simplified assumptions regarding observables and interactions (15, 74, 75) may also enable analysis of cases involving underlying complex dynamics. While realizing both i) and ii) remains challenging, progress in both areas suggests that our decomposition could potentially be extended to analyze experimental data in the future.

## Materials and Methods

This section summarizes the numerical procedures used for the noisy FitzHugh–Nagumo model (Section *Applications to the noisy FitzHugh–Nagumo model*); see also the *SI Appendix*, *Supplementary Methods*. The numerical calculation consists of three steps: i) computing the housekeeping part of the local mean velocity $\nu_{t}^{\text{hk}}$ in the steady state, ii) simulating the virtual dynamics driven by $\nu_{t}^{\text{hk}}$ in Eq. **6**, and iii) extracting Koopman eigenfunctions and modes from the generated time-series data and calculating the terms of our decomposition in Eq. **10**. The parameter settings were as follows. For Fig. 3*A*–*E*, we used a=0, b=0.5, I=0, $T=10^{-3}$, and τ=12.5. For Fig. 3*F*–*H*, the same parameters were used except that τ was varied from 2.5 to 22.5 in increments of 5. For Fig. 4, we set a=0, b=0.5, $T=10^{-3}$, and τ=12.5, while varying I from 0 to 2.5 in increments of 0.1. For Fig. 6, we fixed a=0, b=2, I=0, and τ=12.5, with T varied from $10^{-4}$ to $10^{-1}$ in logarithmic steps of 0.1.

### Calculation of the Local Mean Velocity.

We calculated the housekeeping part of the local mean velocity $\nu_{t}^{\text{hk}}$ from the noisy FitzHugh–Nagumo model in Eq. **13**. The decomposition was performed in the steady state, where the excess part vanishes and $\nu_{t}=\nu_{t}^{\text{hk}}$. To compute $\nu_{t}$, we estimated the steady-state distribution $p_{t}$, satisfying $\frac{\partial }{\partial t}p_{t}(x)$, using the discretization approach of ref. 65: The continuous Langevin equation in Eq. **13** was converted into a discrete transition-rate matrix on a grid. We discretized $x^{\left(1\right)}$ and $x^{\left(2\right)}$ into $10^{4}$ intervals over [−5,5] and [−5+I,5+I], respectively, resulting in $10^{4\times 2}$ grid points. In this discrete system, we constructed the transition-rate matrix corresponding to Eq. **13** and obtained the steady-state distribution from the eigenvector associated with its zero eigenvalue. From this distribution, we computed $\nabla \ln p_{t}$ by cubic-spline interpolation followed by differentiation, allowing us to evaluate the local mean velocity at arbitrary locations.

### Simulating the Virtual Dynamics.

Having obtained $\nu_{t}^{\text{hk}}$, we generated time-series trajectories by simulating the virtual dynamics in Eq. **6**. We used an eighth-order Runge–Kutta method with a time step Δs=1, generating trajectories of length S=150. For each parameter setting, we generated N=1,000 independent trajectories to evaluate the decomposition. These trajectories serve as Monte Carlo samples for approximating the terms in our decomposition, and their initial conditions were sampled from the discretized steady-state distribution $p_{t}\left(x\right)$. Hereafter, $xx_{n,s}$ denotes the state at time s of the n-th trajectory.

### Extraction of Koopman Eigenfunctions and Modes.

From the simulated time-series data, we estimated the Koopman eigenfunctions ${\left\{\phi_{n,k}\right\}}_{k=1}^{r_{n}}$, eigenvalues ${\left\{\lambda_{n,k}\right\}}_{k=1}^{r_{n}}$, and modes ${\left\{v_{n,k}\right\}}_{k=1}^{r_{n}}$ for each trajectory. Because different trajectories have different supports in state space, the eigenfunctions obtained from distinct trajectories were regarded as different functions. Here, $r_{n}$ denotes the number of extracted modes for the n-th trajectory, and the double subscript_*n,k*_ indicates the k-th eigenfunction, eigenvalue, or mode for that trajectory. The number of extracted modes $r_{n}$ varies across trajectories, and the procedure for determining $r_{n}$ is described later. To obtain these quantities, we employed Hankel DMD (54–56) in combination with physics-informed DMD (PiDMD) (58) via the PyDMD Python package (57). Hankel DMD constructs a vector of $h_{n}$ observable functions, $g\left(x\right)={\left(g_{1}{\left(x\right)}^{⊤},g_{2}{\left(x\right)}^{⊤},\cdots ,g_{h_{n}}{\left(x\right)}^{⊤}\right)}^{⊤}={\left(\;\text{Id}{\left(x\right)}^{⊤},{\left(e^{\Delta sK}\;\text{Id}\left(x\right)\right)}^{⊤},\cdots ,{\left(e^{(h_{n}-1)\Delta sK}\;\text{Id}\left(x\right)\right)}^{⊤}\right)}^{⊤}\in R^{dh_{n}}$, where Id(x)=x is the identity function. Here, $h_{n}$ denotes the number of time delays chosen for the n-th trajectory, serving as a hyperparameter of the fitting procedure. The procedure for selecting $h_{n}$ is also described later. This vector is obtained by time-delay embedding of the time-series data, i.e., $g\left(x_{n,s}\right)={\left(x_{n,s}^{⊤},x_{n,s+\Delta s}^{⊤},\cdots ,x_{n,s+(h_{n}-1)\Delta s}^{⊤}\right)}^{⊤}$. Since the Koopman generator K is linear, the time evolution of this observable vector can be approximated by a linear dynamical system, even when the dynamics of $xx_{n,s}$ are nonlinear.

To estimate the Koopman generator K while ensuring that its eigenvalues are purely imaginary, we applied PiDMD, which constrains the representation matrix of $e^{\Delta sK}$ to be unitary during the fitting procedure. Let L denote the representation matrix of $e^{\Delta sK}$. Under stationarity, the covariance matrix Σ satisfies $\Sigma =L\Sigma L^{*}$, implying that L is unitary when Σ is the identity matrix. Therefore, before applying PiDMD, we linearly transformed the delay-embedded data so that its covariance matrix became the identity matrix. Specifically, we centered the delay-embedded observable vectors by subtracting their temporal mean, $\bar{g}=\left(1/\left(S-h_{n}+1\right)\right)\sum_{s=1}^{S-h_{n}+1}g\left(x_{n,s}\right)$, and defined $G=(g\left(x_{n,1}\right)-\bar{g},\;g\left(x_{n,2}\right)-\bar{g},\;\cdots ,\;g\left(x_{n,S-h_{n}+1}\right)-\bar{g})$. We then performed a singular value decomposition $G=USV^{⊤}$, whitened the data as $\tilde{G}=S^{-1}U^{⊤}G$, and applied PiDMD to G to obtain the Koopman modes $\left\{{\tilde{v}}_{n,k}\right\}$. Here, the number of extracted modes $r_{n}$ was determined for each trajectory from this singular value decomposition by applying optimal singular value hard thresholding (SVHT) (76) to the singular values of G. Accordingly, $r_{n}$ varies across trajectories; for the example trajectory shown in Fig. 3 *B* and *C*, we obtained $r_{n}=27$, whereas in analyses combining modes across trajectories, such as Fig. 3*D*, we used all extracted modes, giving a total of $r=\sum_{n=1}^{N}r_{n}$ modes. Finally, the obtained modes were mapped back to the original coordinate system as $v_{n,k}=US{\tilde{v}}_{n,k}$, and the temporal mean $\bar{g}$ was added back to the reconstructed trajectories so that they could be interpreted in the original observable space $g(x_{n,s})$.

For each trajectory, the number of time delays $h_{n}$ was chosen from 1 to 100 in increments of 1 to minimize the reconstruction error based on Eq. **7**, $\sum_{s=1}^{S}{∥x_{n,s}-\sum_{k=1}^{r_{n}}e^{\lambda_{n,k}s}\;\phi_{n,k}\left(x_{n,0}\right)\;v_{n,k}∥}^{2}$, which varies with $h_{n}$ because the extracted eigenvalues $\lambda_{n,k}$, eigenfunctions $\phi_{n,k}$, and modes $v_{n,k}$ depend on the chosen $h_{n}$.

### Computation of the Terms of Our Decomposition.

From the Koopman eigenvalues, eigenfunctions, and modes obtained for each trajectory, we computed the terms of our decomposition as follows:

[14] $$\begin{array}{cc} & \chi_{n,k}=\left|\lambda_{n,k}/\left(2\pi \;\text{i}\right)\right| \end{array}$$

[15] $$\begin{array}{cc} & J_{n,k}=\frac{1}{S}\sum_{s}{\left(\phi_{n,k}\left(x_{n,s}\right)v_{n,k}\right)}^{*}D_{t}^{-1}\left(\phi_{n,k}\left(x_{n,s}\right)v_{n,k}\right), \end{array}$$

[16] $$\begin{array}{cc} & \sigma_{t}^{\text{hk},(n,k)}={\left(2\pi \right)}^{2}\chi_{n,k}^{2}J_{n,k}, \end{array}$$

where the expected values in Eq. **10** are approximated as time-averages within each trajectory. Since each eigenfunction is supported only within its corresponding trajectory and does not overlap with those from other trajectories, the associated quantities $\chi_{n,k}$, $J_{n,k}$, and $\sigma_{t}^{\text{hk},(n,k)}$ were considered as distinct terms for each trajectory. To summarize the results in a form consistent with ensemble expectations, we concatenated these quantities and reindexed them, dividing each trajectory-specific contribution by N: $\left\{\;J_{1,1}/N,...,J_{1,r_{1}}/N,...,J_{N,1}/N,...,J_{N,r_{N}}/N\;\right\}$, $\{\sigma_{t}^{\text{hk},(1,1)}/$$N,...,\sigma_{t}^{\text{hk},(1,r_{1})}/N,...,\sigma_{t}^{\text{hk},(N,1)}/N,...,\sigma_{t}^{\text{hk},(N,r_{N})}/N\}$. These reindexed quan-tities were plotted in Figs. 3–6. In frequency-resolved panels such as Fig. 3*D*, we used a moving window to reduce overlap and density bias across frequencies. Values were summed within bins of width $10^{-3}$ and plotted at the bin centers.

## Supplementary Material

Appendix 01 (PDF)

## Data Availability

The code used to generate the simulation data and to perform the numerical analyses and figure generation has been deposited in Zenodo at https://doi.org/10.5281/zenodo.20496814 (77). All other data are included in the article and/or *SI Appendix*.
