# Supplementary material for: Koopman mode decomposition of thermodynamic dissipation in nonlinear Langevin dynamics
Source: Proc Natl Acad Sci U S A. 2026 Jun 18;123(25):e2530617123. doi: 10.1073/pnas.2530617123 (PMC13291601; doi:10.1073/pnas.2530617123)
Supplement: Supplementary file 1 — Appendix 01 (PDF) [file pnas.2530617123.sapp.pdf]

Supporting Information for

Koopman mode decomposition of thermodynamic dissipation in nonlinear Langevin dynamics

Daiki Sekizawa, Sosuke Ito and Masafumi Oizumi

Daiki Sekizawa
E-mail: sekizawa-daiki963@g.ecc.u-tokyo.ac.jp

This PDF file includes:

Supporting text
Figs. S1 to S3
SI References

Supporting Information Text

Contents

Koopman mode decomposition of virtual dynamics given by $\boldsymbol{\nu}_t^{\mathrm{hk}}$ 2

Skew-adjointness and diagonalizability of the Koopman generator 3

Koopman mode decomposition of the housekeeping entropy production rate 4
- The main result . . . 4
- Derivation of the main result . . . 4

Linear Langevin dynamics 5

Physical meaning of frequencies extracted in our methods 5
- (A) Comparison with frequencies extracted by linear stability analysis . . . 6
- (B) Comparison with the limit-cycle frequency in a small-noise regime . . . 8
- (C) Comparison with frequencies extracted by the stochastic operator . . . 8

Eigenvalues of the Fokker–Planck operator for linear Langevin dynamics 9

Application of our decomposition to non-steady state 10
- Finite sampling effects in low-noise metastable systems . . . 12

Supplementary Methods 12
- Calculation of the local mean velocity . . . 12
- Simulating the virtual dynamics . . . 13
- Extraction of Koopman eigenfunctions and modes . . . 13
- Computation of the terms of our decomposition . . . 13
- Estimation of confidence intervals . . . 14
- Calculation of the true values of the housekeeping entropy production rates . . . 14
- Calculation of correlation times . . . 14
- Comparison with the limit-cycle frequency . . . 15
- Comparison with the frequencies extracted by linear stability analysis . . . 15
- Application of our decomposition to non-steady state dynamics . . . 15

Koopman mode decomposition of virtual dynamics given by $\boldsymbol{\nu}_t^{\mathrm{hk}}$

In this section, we provide a more detailed explanation of how the virtual deterministic process introduced in Eq. 6 can be represented using Koopman mode decomposition. The virtual deterministic process is given by

$$d\boldsymbol{x}_s = \boldsymbol{\nu}_t^{\mathrm{hk}}(\boldsymbol{x}_s)\, ds. \tag{S1}$$

As explained in the main text, the housekeeping part of the local mean velocity $\boldsymbol{\nu}_t^{\mathrm{hk}}(\boldsymbol{x})$ is derived from the original process [Eq. 1] to obtain its housekeeping entropy production rate σ_t^{hk}. In the virtual deterministic process in Eq. S1, the subscript s denotes the virtual time, and t denotes the time of the original Langevin dynamics. In this virtual dynamics, $\boldsymbol{\nu}_t^{\mathrm{hk}}(\boldsymbol{x})$ is fixed with respect to s, and the probability distribution $p_t(\boldsymbol{x})$ of the original dynamics serves as an invariant measure of the virtual dynamics.

The nonlinear dynamics in Eq. S1 can be reformulated as a linear dynamical system in function space by extracting a finite number of modes via Koopman mode decomposition (1, 2) (see Fig. 2 in the main text). In continuous time, this is achieved using the Koopman generator $\mathcal{K}$, which is defined as the infinitesimal generator of the Koopman operator. For any observable $g : \mathbb{R}^d \to \mathbb{C}$, the generator acts as

$$\mathcal{K}g(\boldsymbol{x}) := \nabla g(\boldsymbol{x}) \cdot \boldsymbol{\nu}_t^{\mathrm{hk}}(\boldsymbol{x}). \tag{S2}$$

By definition, it satisfies

$$\mathcal{K}g(\boldsymbol{x}_s) = \nabla g(\boldsymbol{x}_s) \cdot \frac{d\boldsymbol{x}_s}{ds} = \frac{d}{ds} g(\boldsymbol{x}_s), \tag{S3}$$

which describes the time evolution of the observable $g(\boldsymbol{x}_s)$. Moreover, $\mathcal{K}$ is linear. For any observables g_1 and g_2, and scalars a and b, the relation

$$\mathcal{K}(ag_1 + bg_2) = a\mathcal{K}g_1 + b\mathcal{K}g_2, \tag{S4}$$

holds. Thus, the Koopman generator converts the nonlinear dynamics of Eq. S1 into linear evolution in function space. For the identity observable $\mathrm{Id}(\boldsymbol{x}) = \boldsymbol{x}$, its time evolution under Eq. S1 can be written as

$$\boldsymbol{x}_{s+\Delta s} = \mathrm{Id}(\boldsymbol{x}_{s+\Delta s}) = e^{\Delta s \mathcal{K}}\, \mathrm{Id}(\boldsymbol{x}_s). \tag{S5}$$

This equation demonstrates that the nonlinear dynamics of $\boldsymbol{x}$ are represented by a linear dynamical system in function space, with the identity observable $\mathrm{Id}(\cdot)$ as the initial condition.

Using the Koopman eigenfunctions as a basis for the function space enables us to understand that the nonlinear dynamics of $\boldsymbol{x}_s$ can be expressed as a sum of modal contributions in the virtual dynamics. We define the Koopman eigenfunctions $\{\phi_k\}_{k=1}^r$ and eigenvalues $\{\lambda_k\}_{k=1}^r$ as those that satisfy the following:

$$\mathcal{K}\phi_k(\boldsymbol{x}_s) = \lambda_k \phi_k(\boldsymbol{x}_s) = \frac{d}{ds}\phi_k(\boldsymbol{x}_s), \tag{S6}$$

where r denotes the number of eigenvalues. The value of r could be any integer, including infinity. These eigenfunctions are solved as

$$\phi_k(\boldsymbol{x}_{s+\Delta s}) = e^{\lambda_k \Delta s}\phi_k(\boldsymbol{x}_s). \tag{S7}$$

Here, we assume that the number of modes r is finite. Under this condition, as shown in Section *"Skew-adjointness and diagonalizability of the Koopman generator"*, the Koopman generator $\mathcal{K}$ becomes diagonalizable. Using this property, we expand the identity function $\mathrm{Id}(\boldsymbol{x}) = \boldsymbol{x}$ with weight vectors $\{\boldsymbol{v}_k\}_{k=1}^r$ as

$$\mathrm{Id}(\boldsymbol{x}) = \sum_k^r \phi_k(\boldsymbol{x})\boldsymbol{v}_k. \tag{S8}$$

We then obtain the Koopman mode decomposition

$$\boldsymbol{x}_{s+\Delta s} = \sum_k^r e^{\lambda_k \Delta s}\phi_k(\boldsymbol{x}_s)\boldsymbol{v}_k. \tag{S9}$$

The vector $\boldsymbol{v}_k$ is known as the Koopman mode. In this decomposition, the nonlinear dynamics of $\boldsymbol{x}_s$ is understood as a sum of the modal contributions. As the only time-dependent part is $e^{\lambda_k \Delta s}$, the Koopman eigenvalues $\{\lambda_k\}_{k=1}^r$ determine the characteristics of the modal dynamics. The real part of each eigenvalue determines the exponential growth or decay rate, while the imaginary part determines the oscillation frequency. In data analysis, dynamic mode decomposition offers efficient methods for extracting a finite number of modes from time series data.

We define the frequency χ_k as

$$\chi_k = |\lambda_k/(2\pi \mathrm{i})|, \tag{S10}$$

where i stands for the imaginary unit. As will be derived in the next section, the eigenvalue λ_k for Eq. S1 is purely imaginary, and therefore, χ_k is a real number. This implies that the time-variation of $\boldsymbol{x}_s$ in Eq. S9 is expressed as the sum of oscillatory modes.

We also introduce the intensities of the oscillatory modes. When the eigenvalues are not degenerate, the intensity of the k-th oscillatory mode is given by

$$J_k = \langle (\phi_k \boldsymbol{v}_k)^* D_t^{-1} (\phi_k \boldsymbol{v}_k) \rangle_t. \tag{S11}$$

The symbol * stands for conjugate transpose. This quantity is the L2-norm of the k-th mode $\phi_k \boldsymbol{v}_k$ under the metric $D_t^{-1} p_t(\boldsymbol{x})$. Therefore, it represents the intensity of the k-th oscillatory mode.

Skew-adjointness and diagonalizability of the Koopman generator

To discuss the diagonalizability of the Koopman generator, we first examine its adjoint property in the context of the virtual dynamics driven by the housekeeping local mean velocity $\boldsymbol{\nu}_t^{\mathrm{hk}}(\boldsymbol{x})$. In this system, where $\nabla \cdot (\boldsymbol{\nu}_t^{\mathrm{hk}}(\boldsymbol{x}) p_t(\boldsymbol{x})) = 0$ holds, the Koopman generator $\mathcal{K}$ is a skew-adjoint operator, although this property does not hold for general dynamical systems. For any functions $g_1(\boldsymbol{x})$ and $g_2(\boldsymbol{x})$, we can calculate $\langle g_2 \mathcal{K} g_1 \rangle_t = \int d\boldsymbol{x} g_2(\boldsymbol{x}) p_t(\boldsymbol{x}) \mathcal{K} g_1(\boldsymbol{x})$ as

$$\begin{aligned}
\langle g_2 \mathcal{K} g_1 \rangle_t &= \int d\boldsymbol{x} g_2(\boldsymbol{x}) p_t(\boldsymbol{x}) \nabla g_1(\boldsymbol{x}) \cdot \boldsymbol{\nu}_t^{\mathrm{hk}}(\boldsymbol{x}) \\
&= -\int d\boldsymbol{x} g_1(\boldsymbol{x}) g_2(\boldsymbol{x}) \nabla \cdot (\boldsymbol{\nu}_t^{\mathrm{hk}}(\boldsymbol{x}) p_t(\boldsymbol{x})) - \int d\boldsymbol{x} g_1(\boldsymbol{x}) p_t(\boldsymbol{x}) \nabla g_2(\boldsymbol{x}) \cdot \boldsymbol{\nu}_t^{\mathrm{hk}}(\boldsymbol{x}) \\
&= -\langle g_1 \mathcal{K} g_2 \rangle_t,
\end{aligned} \tag{S12}$$

where we used $\nabla \cdot (\boldsymbol{\nu}_t^{\mathrm{hk}}(\boldsymbol{x}) p_t(\boldsymbol{x})) = 0$ and applied integration by parts assuming that the distribution $p_t(\boldsymbol{x})$ becomes zero at infinity. Therefore, the relation $\langle g_2 \mathcal{K} g_1 \rangle_t = -\langle g_1 \mathcal{K} g_2 \rangle_t$ shows that the Koopman generator is a skew-adjoint operator. This result implies that the matrix corresponding to $p_t(\boldsymbol{x})\mathcal{K}$ is antisymmetric when the Koopman generator is approximated numerically as a finite-dimensional matrix. Accordingly, the Koopman generator $\mathcal{K}$ is diagonalizable if the finite-dimensional approximation is sufficiently accurate.

Koopman mode decomposition of the housekeeping entropy production rate

The main result. As mentioned in the main text, our main result is a decomposition of the housekeeping entropy production rate into independent positive contributions from each oscillatory mode:

$$\sigma_t^{\mathrm{hk}} = \sum_k^r \sigma_t^{\mathrm{hk},(k)}$$
$$\sigma_t^{\mathrm{hk},(k)} = (2\pi)^2 \chi_k^2 J_k. \qquad \text{[S13]}$$

The decomposition means that the contribution of each oscillatory mode to the housekeeping entropy production rate is the product of its frequency squared χ_k^2 and its intensity J_k (see also Fig. 2b). In other words, modes with higher frequencies and greater intensities have a greater impact on the housekeeping entropy production rate.

When the eigenvalues are degenerate, the decomposition becomes

$$\sigma_t^{\mathrm{hk}} = \sum_{k'} (2\pi)^2 \chi_{k'}^2 \left\langle \left(\sum_{l \in C_{k'}} \phi_l \boldsymbol{v}_l \right)^* D_t^{-1} \left(\sum_{m \in C_{k'}} \phi_m \boldsymbol{v}_m \right) \right\rangle_t , \qquad \text{[S14]}$$

where $C_{k'} := \{ l \mid \lambda_l = 2\pi \chi_{k'} \mathrm{i} \}$ is the set of indices corresponding to the degenerate eigenvalue $2\pi i \chi'_k$. Here, the index k' is defined such that each $\chi_{k'}$ is distinct. The summation is taken over only those k' for which $\chi_{k'}$ has different values, ensuring that no k' with the same value of $\chi_{k'}$ is selected more than once.

Derivation of the main result. We derive the main result [Eq. S13] using the Koopman mode decomposition, and the fact that the Koopman eigenvalues for the virtual dynamics in Eq. S1 are purely imaginary.

Using the Koopman mode decomposition [Eq. S9], the housekeeping local mean velocity can be expressed as

$$\boldsymbol{\nu}_t^{\mathrm{hk}}(\boldsymbol{x}_s) = \frac{d\boldsymbol{x}_s}{ds} = \sum_{k=1}^r \lambda_k \phi_k(\boldsymbol{x}_s) \boldsymbol{v}_k. \qquad \text{[S15]}$$

The housekeeping entropy production rate σ_t^{hk} in the original dynamics [Eq. 1] is then calculated as

$$\begin{aligned} \sigma_t^{\mathrm{hk}} &= \left\langle \left(\boldsymbol{\nu}_t^{\mathrm{hk}}\right)^* D_t^{-1} \boldsymbol{\nu}_t^{\mathrm{hk}} \right\rangle_t \\ &= \left\langle \left(\sum_k \lambda_k \phi_k \boldsymbol{v}_k \right)^* D_t^{-1} \left(\sum_l \lambda_l \phi_l \boldsymbol{v}_l \right) \right\rangle_t \\ &= \sum_{k,l} \lambda_k^* \lambda_l \left\langle (\phi_k \boldsymbol{v}_k)^* D_t^{-1} (\phi_l \boldsymbol{v}_l) \right\rangle_t . \end{aligned} \qquad \text{[S16]}$$

Here, the symbol $*$ is also used to represent the complex conjugate when applied to scalars. First, we show that the Koopman eigenfunctions are orthogonal, i.e., $\langle \phi_k^* \phi_l \rangle_t = 0$ if $\lambda_k \neq \lambda_l$. This orthogonality transforms Eq. S16 into our main result [Eq. S13]. To prove this orthogonality, we consider the following identity:

$$(\lambda_k^* + \lambda_l) \langle \phi_k^* \phi_l \rangle_t = (\lambda_k^* + \lambda_l) \int d\boldsymbol{x}_s \; p_t(\boldsymbol{x}_s) \phi_k(\boldsymbol{x}_s)^* \phi_l(\boldsymbol{x}_s) \qquad \text{[S17]}$$

$$\begin{aligned} &= \int d\boldsymbol{x}_s \; p_t(\boldsymbol{x}_s) \; \frac{d}{ds} (\phi_k(\boldsymbol{x}_s)^* \phi_l(\boldsymbol{x}_s)) \\ &= \int d\boldsymbol{x}_s \; p_t(\boldsymbol{x}_s) \; \boldsymbol{\nu}_t^{\mathrm{hk}} \cdot \nabla (\phi_k(\boldsymbol{x}_s)^* \phi_l(\boldsymbol{x}_s)) \\ &= - \int d\boldsymbol{x}_s \; [\nabla \cdot (p_t(\boldsymbol{x}_s) \boldsymbol{\nu}_t^{\mathrm{hk}})] (\phi_k(\boldsymbol{x}_s)^* \phi_l(\boldsymbol{x}_s)) \\ &= 0, \end{aligned} \qquad \text{[S18]}$$

where we applied integration by parts and used the definition of the Koopman generator [Eq. S2] and the definition of the housekeeping local mean velocity, i.e., $0 = -\nabla \cdot [\boldsymbol{\nu}_t^{\mathrm{hk}}(\boldsymbol{x}) p_t(\boldsymbol{x})]$. From this identity [Eq. S18], we obtain $\langle \phi_k^* \phi_l \rangle_t = 0$ when $\lambda_k \neq \lambda_l$.

From this identity [Eq. S18], we can also prove that the Koopman eigenvalues $\{\lambda_k\}_{k=1}^r$ are purely imaginary. Substituting $k = l$ into Eq. S18 yields $\lambda_k^* + \lambda_k = 0$, since $\langle |\phi_k|^2 \rangle_t > 0$. Therefore, all the eigenvalues are purely imaginary.

Linear Langevin dynamics

From our main result [Eq. S13], we can derive the decomposition for the linear Langevin process as a special case. This special case was obtained in our previous work (3).

We consider the following linear Langevin process:

$$d\boldsymbol{x}_t = D_t A_t \boldsymbol{x}_t \, dt + \sqrt{2D_t} \, d\boldsymbol{B}_t. \tag{S19}$$

Here, A_t is a matrix representing the linear dynamics. We assume that the distribution $p_t(\boldsymbol{x})$ is Gaussian. In order to apply our decomposition, we consider the virtual dynamics

$$d\boldsymbol{x}_s = \boldsymbol{\nu}_t^{\mathrm{hk}}(\boldsymbol{x}_s) \, ds = D_t A_t^{\mathrm{hk}} \, \boldsymbol{x}_s \, ds, \tag{S20}$$

where A_t^{hk} is a matrix discussed in Ref. (3). The matrix A_t^{hk} exists if the original Langevin process in Eq. S19 is linear and if the distribution $p_t(\boldsymbol{x})$ is Gaussian. We note that the real matrix $D_t A_t^{\mathrm{hk}}$ can be expressed as the product of a real antisymmetric matrix and a positive-definite symmetric matrix (see Ref. (3)), and is diagonalizable. Because $D_t A_t^{\mathrm{hk}}$ is diagonalizable, this virtual dynamics is solved as follows:

$$\boldsymbol{x}_{s+\Delta s} = e^{D_t A_t^{\mathrm{hk}} \Delta s} \, \boldsymbol{x}_s \tag{S21}$$

$$= \sum_k e^{\lambda_k \Delta s} \, \mathsf{P} \boldsymbol{e}_k \boldsymbol{e}_k^\top \mathsf{P}^{-1} \boldsymbol{x}_s \tag{S22}$$

$$= \sum_k e^{\lambda_k \Delta s} \, \mathsf{F}_k \, \boldsymbol{x}_s, \tag{S23}$$

where λ_k is the k-th eigenvalue of $D_t A_t^{\mathrm{hk}}$ and we consider the eigendecomposition of $D_t A_t^{\mathrm{hk}}$:

$$D_t A_t^{\mathrm{hk}} = \mathsf{P} \Lambda \mathsf{P}^{-1} = \sum_k \lambda_k \, \mathsf{P} \boldsymbol{e}_k \boldsymbol{e}_k^\top \mathsf{P}^{-1} = \sum_k \lambda_k \, \mathsf{F}_k, \tag{S24}$$

$$\mathsf{F}_k = \mathsf{P} \boldsymbol{e}_k \boldsymbol{e}_k^\top \mathsf{P}^{-1}. \tag{S25}$$

The matrix P is regular and complex-valued. The matrix F_k is regarded as the projection matrix. The matrix Λ is a diagonal matrix with k-th *entry* being the k-th eigenvalue λ_k. The vector $\boldsymbol{e}_k$ has a value of 1 in the k-th position and 0 in all other positions.

We can relate this solution to the expression in Eq. S9, which uses Koopman eigenfunctions and modes, by considering the following quantities:

$$\phi_k(\boldsymbol{x}) = \boldsymbol{e}_k^\top \mathsf{P}^{-1} \boldsymbol{x}, \tag{S26}$$

$$\boldsymbol{v}_k = \mathsf{P} \boldsymbol{e}_k. \tag{S27}$$

Because the Koopman generator $\mathcal{K}$ is given by $\mathcal{K} g(\boldsymbol{x}) = \nabla g(\boldsymbol{x}) \cdot \boldsymbol{\nu}_t^{\mathrm{hk}}(\boldsymbol{x}) = \nabla g(\boldsymbol{x}) \cdot D_t A_t^{\mathrm{hk}} \, \boldsymbol{x}$, we obtain $\mathcal{K} \phi_k(\boldsymbol{x}) = ((\mathsf{P}^{-1})^\top \boldsymbol{e}_k) \cdot \mathsf{P} \Lambda \mathsf{P}^{-1} \boldsymbol{x} = \lambda_k \boldsymbol{e}_k^\top \mathsf{P}^{-1} \boldsymbol{x} = \lambda_k \phi_k(\boldsymbol{x})$. Therefore, the k-th eigenvalue λ_k of the matrix $D_t A_t^{\mathrm{hk}}$ is regarded as the k-th Koopman eigenvalue. By substituting the eigenvalue λ_k, eigenfunction ϕ_k, and Koopman mode $\boldsymbol{v}_k$ into our decomposition in Eq. S13, we obtain the result presented in Ref. (3) as a special case:

$$\begin{aligned} \sigma_t^{\mathrm{hk}} &= \sum_k |\lambda_k|^2 \left\langle (\phi_k \boldsymbol{v}_k)^* D_t^{-1} (\phi_k \boldsymbol{v}_k) \right\rangle_t \\ &= \sum_k |\lambda_k|^2 \left\langle ((\boldsymbol{e}_k^\top \mathsf{P}^{-1} \boldsymbol{x}_t)(\mathsf{P} \boldsymbol{e}_k))^* D_t^{-1} ((\boldsymbol{e}_k^\top \mathsf{P}^{-1} \boldsymbol{x}_t)(\mathsf{P} \boldsymbol{e}_k)) \right\rangle_t \\ &= \sum_k |\lambda_k|^2 \left\langle (\mathsf{F}_k \boldsymbol{x}_t)^* D_t^{-1} (\mathsf{F}_k \boldsymbol{x}_t) \right\rangle_t. \end{aligned} \tag{S28}$$

Physical meaning of frequencies extracted in our methods

In this section, we further examine the oscillatory frequencies extracted by our decomposition in order to clarify their physical meaning. Specifically, we compare the frequencies extracted by our decomposition with three established notions of oscillation in dynamical systems: (A) the characteristic frequency predicted by linear stability analysis around a stable fixed point, (B) the oscillation frequency of a deterministic limit cycle, and (C) the frequencies associated with the spectrum of the corresponding stochastic (Fokker–Planck) operator.

A common point underlying all three comparisons is that the frequencies extracted by our decomposition are determined not by the deterministic drift $D_t \boldsymbol{f}_t(\boldsymbol{x})$ alone, which governs the zero-noise dynamics, but by the local mean velocity field

$$\boldsymbol{\nu}_t(\boldsymbol{x}) = D_t \boldsymbol{f}_t(\boldsymbol{x}) - D_t \nabla \ln p_t(\boldsymbol{x}), \tag{S29}$$

which includes both the deterministic drift and the diffusion-induced probability transport. Accordingly, as long as the noise is nonzero, even if it is arbitrarily small, the extracted frequencies should be interpreted as frequencies of the stochastic probability flow described by $\boldsymbol{\nu}_t$. This also implies that their relation to the oscillatory structure of the zero-noise deterministic dynamics is not universal, but depends on the situation.

For the comparisons in (A) and (B), we use the noisy FitzHugh–Nagumo model with parameter regimes chosen based on the eigenvalues of the Jacobian of the deterministic drift $D_t\boldsymbol{f}_t$. Figure S1a shows how these eigenvalues change as the parameter I varies. Based on this analysis, we select representative parameter regimes in which the deterministic dynamics either exhibit a limit cycle or admit a stable fixed point, as illustrated in Fig. S1. For (C), we consider the linear Langevin setting, where the relationship to the stochastic generator spectrum can be stated explicitly. The analysis methods used in this section are described in Section *"Supplementary Methods"*.

The results can be summarized as follows. Around a stable fixed point (A), the characteristic frequency relevant to our decomposition is determined not by the Jacobian of the deterministic drift $D_t\boldsymbol{f}_t$ alone, but by that of the local mean velocity field $\boldsymbol{\nu}_t^{\mathrm{hk}}$, which incorporates the effect of stochastic diffusion through the probability distribution. Accordingly, the frequencies predicted from the Jacobian of $\boldsymbol{\nu}_t^{\mathrm{hk}}$ agree with those extracted by our decomposition. In the limit-cycle regime (B), the dominant frequency extracted by our decomposition coincides with the oscillation frequency of the stochastic dynamics in the representative small-noise regime considered here. Based on these observations, we then discuss in (C) how the frequencies extracted by our decomposition relate to those associated with the stochastic (Fokker–Planck) operator. Since the spectral frequencies of the Fokker–Planck operator are determined by the deterministic drift $D_t\boldsymbol{f}_t$, they do not necessarily coincide with those extracted by our decomposition.

(A) Comparison with frequencies extracted by linear stability analysis. We next examine parameter regimes in which the deterministic drift admits a stable fixed point. In such regimes, and for sufficiently small noise, the dynamics are restricted to fluctuations around the fixed point. As shown in Fig. S1a, we consider two representative cases: one in which the Jacobian

$$J := \left.\frac{\partial(D_t\boldsymbol{f}_t)}{\partial\boldsymbol{x}}\right|_{\boldsymbol{x}=\boldsymbol{x}_{\mathrm{dfix}}} \qquad \text{[S30]}$$

evaluated at the deterministic fixed point $\boldsymbol{x}_{\mathrm{dfix}}$ has a complex conjugate pair of eigenvalues with negative real parts ($I = 1.5$), and another in which all eigenvalues are purely real and negative ($I = 2.4$). Here, the deterministic fixed point $\boldsymbol{x}_{\mathrm{dfix}}$ is defined as $D_t\boldsymbol{f}_t(\boldsymbol{x}_{\mathrm{dfix}}) = \boldsymbol{0}$.

The results show that, in these fixed-point regimes, the frequencies extracted by our decomposition are consistently explained by the linear stability analysis of the local mean velocity field $\boldsymbol{\nu}_t^{\mathrm{hk}}$, whereas agreement with the corresponding analysis of the deterministic drift occurs only in some cases. To make this point explicit, we compare the two representative fixed-point regimes introduced above. In (A-1), the Jacobian of the deterministic drift already has complex eigenvalues, so the deterministic dynamics contain a damped oscillatory tendency. In this case, the imaginary part of the eigenvalues of the Jacobian of $D_t\boldsymbol{f}_t(\boldsymbol{x})$ happens to agree with the dominant frequency extracted by our decomposition, and the frequency predicted from the Jacobian of the local mean velocity field $\boldsymbol{\nu}_t^{\mathrm{hk}}$ is also consistent with our decomposition. In (A-2), by contrast, the Jacobian of $D_t\boldsymbol{f}_t(\boldsymbol{x})$ has only real eigenvalues, so at the level of the deterministic drift there is no oscillatory frequency to compare with. Nevertheless, in both regimes, the Jacobian of the local mean velocity field $\boldsymbol{\nu}_t^{\mathrm{hk}}$ has imaginary eigenvalues, and the corresponding predicted frequencies are consistent with those extracted by our decomposition. Thus, in this noisy FitzHugh–Nagumo example, our decomposition does not characterize oscillations of the deterministic drift alone, but rather oscillations of the effective probability flow that includes diffusion-induced effects.

(A-1) Stable oscillatory fixed point. We first consider the case $I = 1.5$, and show that even though the deterministic drift $D_t\boldsymbol{f}_t(\boldsymbol{x})$ around the stable fixed point has only a damped oscillatory tendency, the stochastic dynamics exhibit an oscillation that remains sustained around the fixed point by diffusion-induced effects. In this regime, the deterministic drift $D_t\boldsymbol{f}_t(\boldsymbol{x})$ has a stable fixed point with a complex-conjugate pair of Jacobian eigenvalues. Consistently, the Langevin trajectory exhibits stochastic oscillations (Fig. S1e), the power spectral density shows a clear peak (Fig. S1f), and the virtual dynamics generated by $\boldsymbol{\nu}_t^{\mathrm{hk}}$ also exhibit sustained oscillations with the same frequency (Fig. S1g).

The velocity fields clarify why such oscillatory motion persists in the stochastic dynamics even when the noise is arbitrarily small but nonzero. As shown in Fig. S1h, the deterministic drift $D_t\boldsymbol{f}_t(\boldsymbol{x})$ alone drives trajectories toward the stable fixed point and therefore does not by itself sustain oscillation, whereas the diffusion-induced term $-D_t\nabla\ln p_t(\boldsymbol{x})$ modifies the effective velocity field so that the resulting local mean velocity field $\boldsymbol{\nu}_t^{\mathrm{hk}}$ exhibits a circulating probability flow around the fixed point. Figure S1i shows that the Jacobian of $\boldsymbol{\nu}_t^{\mathrm{hk}}$ retains an oscillatory frequency even though the deterministic drift still drives trajectories toward the stable fixed point.

The frequency extracted by our decomposition should therefore be compared with the Jacobian of $\boldsymbol{\nu}_t^{\mathrm{hk}}$ rather than with that of the deterministic drift alone. As shown in Fig. S1j, the oscillation frequency predicted from the imaginary part of the eigenvalues of the Jacobian of $\boldsymbol{\nu}_t^{\mathrm{hk}}$ agrees with the dominant frequency extracted by our decomposition. In this regime, the Jacobian of $\boldsymbol{\nu}_t^{\mathrm{hk}}$ is close to that of the deterministic drift, which is why the frequency predicted from $D_t\boldsymbol{f}_t(\boldsymbol{x})$ also happens to take a similar value. Thus, even in this case, the relevant oscillation extracted by our decomposition is most naturally interpreted as that of the effective probability flow described by $\boldsymbol{\nu}_t^{\mathrm{hk}}$, while the agreement with the deterministic drift is only incidental. This distinction becomes fully explicit in (A-2), where the deterministic drift no longer provides any oscillatory prediction.

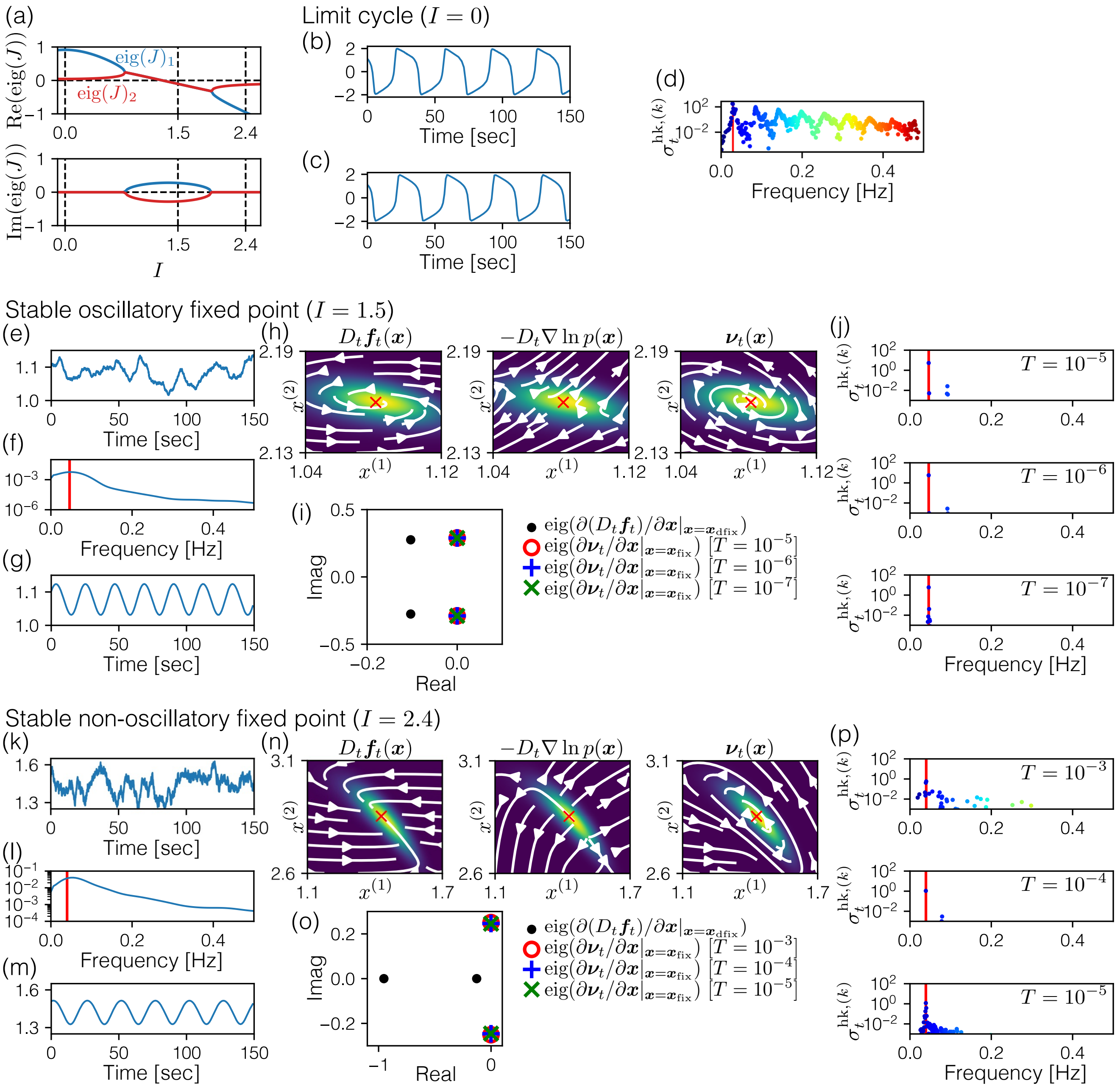

Fig. S1. Comparison between frequencies extracted by our decomposition and those characterized by limit-cycle dynamics and linear stability analysis. (a) Eigenvalues of the Jacobian of the deterministic drift $D_t \boldsymbol{f}_t(\boldsymbol{x})$ evaluated at the fixed point $\boldsymbol{x}_{\rm fix}$ as a function of the input current I, used to select representative parameter regimes: a limit-cycle regime ($I = 0$), a stable oscillatory fixed point ($I = 1.5$), and a stable non-oscillatory fixed point ($I = 2.4$). (b,c) Time series of $x_t^{(1)}$ obtained from the Langevin dynamics (b) and the corresponding virtual dynamics driven by $\boldsymbol{\nu}_t^{\rm hk}$ (c) in the limit-cycle regime ($I = 0$). (d) Frequency-resolved contributions to the entropy production rate obtained by our decomposition for $I = 0$; the red vertical line indicates the limit cycle frequency $f_{\rm LC}$, which is defined in Section *"Supplementary Methods"*. (e-j) Stable oscillatory fixed-point regime ($I = 1.5$). (e) Time series of $x_t^{(1)}$ from the Langevin dynamics. (f) Power spectral density of the time series in (e), estimated using Welch's method; the red vertical line indicates the frequency given by the imaginary part of the Jacobian eigenvalues of $\boldsymbol{\nu}_t^{\rm hk}$. (g) Time series generated by the virtual dynamics. (h) Vector fields of the deterministic drift $D_t \boldsymbol{f}_t(\boldsymbol{x})$ (left), the diffusion-induced term $-D_t \nabla \ln p_t(\boldsymbol{x})$ (middle), and the local mean velocity field $\boldsymbol{\nu}_t^{\rm hk}$ (right); color indicates the steady-state probability density $p_t(\boldsymbol{x})$, and the red cross marks the deterministic fixed point of $D_t \boldsymbol{f}_t$. (i) Eigenvalues of the Jacobian of $D_t \boldsymbol{f}_t(\boldsymbol{x})$ at the deterministic fixed point $\boldsymbol{x}_{\rm dfix}$ and the Jacobian of $\boldsymbol{\nu}_t^{\rm hk}$ at the fixed point $\boldsymbol{x}_{\rm fix}$ for different noise intensities. (j) Frequency-resolved contributions to the entropy production rate. The red vertical line represents the frequency $f_{{\rm LSA},\boldsymbol{\nu}_t}$, which is determined from the linear stability analysis of $\boldsymbol{\nu}_t^{\rm hk}$ and defined in Section *"Supplementary Methods"*. (k-p) Stable non-oscillatory fixed-point regime ($I = 2.4$), shown in the same format as in (e-j).

(A-2) Stable non-oscillatory fixed point. We next consider the case $I = 2.4$, and show more sharply that even though the deterministic drift $D_t \boldsymbol{f}_t(\boldsymbol{x})$ around the stable fixed point has no oscillatory tendency at all, the oscillation extracted by our decomposition remains sustained around the fixed point by diffusion-induced effects. In this regime, the Jacobian of the deterministic drift $D_t \boldsymbol{f}_t(\boldsymbol{x})$ has only real negative eigenvalues, so at the level of the deterministic drift there is no oscillatory frequency to compare with. Nevertheless, the Langevin trajectory exhibits stochastic oscillations (Fig. S1k), the power spectral density shows a clear peak (Fig. S1l), and the virtual dynamics generated by $\boldsymbol{\nu}_t^{\mathrm{hk}}$ also exhibit sustained oscillations with the same frequency (Fig. S1m).

The velocity fields clarify why such oscillatory motion persists in the stochastic dynamics. As shown in Fig. S1n, the deterministic drift $D_t \boldsymbol{f}_t(\boldsymbol{x})$ still drives trajectories toward the stable fixed point, but the relaxation occurs along curved paths in phase space because different directions relax at different rates. When stochastic diffusion is present, random perturbations repeatedly displace the state across these curved deterministic flows, and the diffusion-induced term $-D_t \nabla \ln p_t(\boldsymbol{x})$ modifies the effective velocity field so that the resulting local mean velocity field $\boldsymbol{\nu}_t^{\mathrm{hk}}$ exhibits a circulating probability flow around the fixed point. Figure S1o shows that the Jacobian of $\boldsymbol{\nu}_t^{\mathrm{hk}}$ acquires an oscillatory frequency even though the Jacobian of the deterministic drift has only real eigenvalues.

This distinction persists even in the infinitesimal-noise limit because the diffusion-induced contribution does not disappear from the Jacobian of the effective probability flow. Around the fixed point $\boldsymbol{x}_{\mathrm{fix}}$, the local mean velocity field is approximated by

$$\boldsymbol{\nu}_t(\boldsymbol{x}) \simeq (J + D_t \Sigma^{-1})(\boldsymbol{x} - \boldsymbol{x}_{\mathrm{fix}}), \tag{S31}$$

where J is the Jacobian of the deterministic drift and Σ is the covariance matrix of the stationary distribution near the fixed point. For linear Langevin dynamics, Σ satisfies

$$J\Sigma + \Sigma J^{\top} + 2D_t = 0. \tag{S32}$$

Since Σ scales proportionally to the noise intensity, the product $D_t \Sigma^{-1}$ remains finite even when the noise intensity becomes arbitrarily small. Therefore, even though the Jacobian of the deterministic drift has only real eigenvalues, the Jacobian of $\boldsymbol{\nu}_t$ can acquire imaginary eigenvalues.

The frequency extracted by our decomposition should therefore be compared with the Jacobian of $\boldsymbol{\nu}_t^{\mathrm{hk}}$ rather than with that of the deterministic drift alone. Because the Jacobian of $D_t \boldsymbol{f}_t(\boldsymbol{x})$ has no imaginary part, the deterministic drift cannot explain the nonzero frequency extracted by our decomposition in this regime. By contrast, Fig. S1p shows that the oscillation frequency predicted from the imaginary part of the eigenvalues of the Jacobian of $\boldsymbol{\nu}_t^{\mathrm{hk}}$ agrees with the dominant frequency extracted by our decomposition. Thus, even more clearly than in (A-1), the relevant oscillation extracted by our decomposition is most naturally interpreted as that of the effective probability flow described by $\boldsymbol{\nu}_t^{\mathrm{hk}}$, while the deterministic drift provides no corresponding oscillatory prediction.

(B) Comparison with the limit-cycle frequency in a small-noise regime. We next consider how the frequencies extracted by our decomposition compare with the actual oscillation frequency in the limit-cycle regime. As shown in Fig. S1a, we select the representative limit-cycle regime at $I = 0$. In this regime, simulations of the Langevin dynamics exhibit clear nonlinear oscillations (Fig. S1b). Because the noise intensity is small, the corresponding virtual dynamics driven by the local mean velocity field $\boldsymbol{\nu}_t^{\mathrm{hk}}$ also display oscillatory behavior with a similar characteristic frequency (Fig. S1c).

We then compare the oscillation frequency extracted from the Langevin time series with the frequency-resolved decomposition of the entropy production rate. As shown in Fig. S1d, the dominant contribution to the entropy production rate appears at the same frequency as the oscillation frequency of the Langevin limit cycle. Thus, in the representative small-noise limit-cycle regime considered here, the dominant frequency extracted by our decomposition agrees with the actual oscillation frequency of the stochastic dynamics.

(C) Comparison with frequencies extracted by the stochastic operator. We finally discuss how the frequencies extracted by our decomposition relate to those associated with the stochastic (Fokker–Planck) operator. Based on the fixed-point analyses in Section (A), this comparison can be stated explicitly in the linear Langevin setting. In this setting, the frequencies associated with the Fokker–Planck operator are determined by the deterministic drift $D_t \boldsymbol{f}_t$, whereas the frequencies extracted by our decomposition are determined by the local mean velocity field $\boldsymbol{\nu}_t^{\mathrm{hk}}$. Accordingly, as already seen in Section (A), the two can coincide in some situations, but they need not do so in general, because diffusion-induced effects can modify the effective probability flow and thereby shift the frequencies extracted by our decomposition away from those associated with the deterministic drift. Beyond this linear setting, we do not currently know a universal relation in general nonlinear systems.

The eigenvalues of the Fokker–Planck operator are often used to characterize oscillatory behavior in stochastic systems because the time evolution of the probability density can be expanded in terms of its eigenfunctions. To examine this relation, consider the linearized Langevin dynamics around a stable fixed point,

$$d\boldsymbol{x} = J(\boldsymbol{x} - \boldsymbol{x}_{\mathrm{fix}})\, dt + \sqrt{2D_t}\, d\boldsymbol{B}_t. \tag{S33}$$

If $\mu_1, \ldots, \mu_d$ denote the eigenvalues of the Jacobian J in a d-dimensional system, then the spectrum of the corresponding Fokker–Planck operator $\mathcal{L}_{\mathrm{FP}}$ is given by finite sums of the eigenvalues of J as discussed in Section *"Eigenvalues of the Fokker–Planck operator for linear Langevin dynamics"*. When the fixed point is stable so that $\mathrm{Re}(\mu_i) \leq 0$ with equality only for the stationary mode, the eigenvalues whose real parts are closest to zero correspond directly to the eigenvalues μ_i themselves.

The imaginary parts of these eigenvalues therefore determine the frequency of the most persistent oscillatory component of the stochastic dynamics.

By contrast, the frequencies extracted by our decomposition reflect oscillatory modes of the effective probability flow including the diffusion-induced contribution. As discussed in Section (A), these oscillatory modes are associated with the Jacobian of the local mean velocity field $\boldsymbol{\nu}_t^{\mathrm{hk}}$, which can differ from that of the deterministic drift. Therefore, even within the linear regime, the relation between the frequencies extracted by our decomposition and those associated with the stochastic generator is not one of universal equality, but is controlled by the difference between $\boldsymbol{\nu}_t^{\mathrm{hk}}$ and $D_t\boldsymbol{f}_t$. When the Jacobians of $\boldsymbol{\nu}_t^{\mathrm{hk}}$ and $D_t\boldsymbol{f}_t$ are close, the two frequencies are similar. When diffusion-induced effects substantially modify the effective velocity field, they differ.

Taken together, these results provide the following physical interpretation of the frequencies extracted by our decomposition. In the limit-cycle regime considered here, the dominant frequency extracted by our decomposition agrees with the actual oscillation frequency of the stochastic dynamics. In the fixed-point regimes considered here, the extracted frequencies represent oscillatory motions of the stochastic probability flow sustained by diffusion-induced effects around the stable fixed point, and are consistently predicted by the linear stability analysis of $\boldsymbol{\nu}_t^{\mathrm{hk}}$. In the linear setting, the comparison with the Fokker–Planck operator further shows that these frequencies are tied to the effective probability flow rather than, in general, to the deterministic drift alone.

Eigenvalues of the Fokker–Planck operator for linear Langevin dynamics

In this section, to help clarify the relation between the stochastic operator and our decomposition in Section *"Physical meaning of frequencies extracted in our methods,"* we provide an intuitive demonstration of the association between the eigenvalues of the Fokker–Planck operator and linear Langevin dynamics. For a detailed proof and explanations, see Refs. (4, 5).

Consider

$$d\boldsymbol{x}_t = A\boldsymbol{x}_t\,dt + \sqrt{2D}\,d\boldsymbol{B}_t, \tag{S34}$$

where $A \in \mathbb{R}^{d\times d}$, $D \in \mathbb{R}^{d\times d}$ is symmetric positive semidefinite, and $\boldsymbol{B}_t$ is a d-dimensional standard Brownian motion. The corresponding Fokker–Planck operator $\mathcal{L}_{\mathrm{FP}}$ is defined as

$$\mathcal{L}_{\mathrm{FP}} p_t = -\sum_i \partial_{x_i}\left(\sum_j A_{ij}x_j\,p_t\right) + \sum_{i,j}\partial_{x_i}\left(D_{ij}\partial_{x_j}p_t\right). \tag{S35}$$

If all eigenvalues of A have negative real parts, then the process admits a stationary Gaussian density

$$\rho_{\mathrm{ss}}(\boldsymbol{x}) = \frac{1}{Z}\exp\left(-\frac{1}{2}\boldsymbol{x}^\top\Sigma^{-1}\boldsymbol{x}\right), \tag{S36}$$

where Σ satisfies

$$A\Sigma + \Sigma A^\top + 2D = 0. \tag{S37}$$

The key simplification is that $\mathcal{L}_{\mathrm{FP}}$ preserves the form $P\rho_{\mathrm{ss}}$, where P is a polynomial in $\boldsymbol{x}$. Indeed, using $\nabla\rho_{\mathrm{ss}} = -\Sigma^{-1}\boldsymbol{x}\rho_{\mathrm{ss}}$ and $\mathcal{L}_{\mathrm{FP}}\rho_{\mathrm{ss}} = 0$, one finds

$$\mathcal{L}_{\mathrm{FP}}(P\rho_{\mathrm{ss}}) = \rho_{\mathrm{ss}}\left((\tilde{A}\boldsymbol{x})\cdot\nabla P + \sum_{i,j}D_{ij}\partial_{x_i}\partial_{x_j}P\right), \tag{S38}$$

where

$$\tilde{A} := \Sigma A^\top\Sigma^{-1}. \tag{S39}$$

Here, $\tilde{A}$ has the same eigenvalues as A.

We first construct eigenfunctions corresponding to the eigenvalues of A. For simplicity, assume that A is diagonalizable over $\mathbb{C}$, and let $\boldsymbol{v}_i$ satisfy

$$A\boldsymbol{v}_i = \mu_i\boldsymbol{v}_i. \tag{S40}$$

We introduce the linear functions

$$y_i(\boldsymbol{x}) := \boldsymbol{v}_i^\top\Sigma^{-1}\boldsymbol{x}, \tag{S41}$$

as a polynomial P. Then, since $\nabla y_i = \Sigma^{-1}\boldsymbol{v}_i$,

$$(\tilde{A}\boldsymbol{x})\cdot\nabla y_i = \boldsymbol{x}^\top\tilde{A}^\top\Sigma^{-1}\boldsymbol{v}_i = \boldsymbol{x}^\top\Sigma^{-1}A\boldsymbol{v}_i = \mu_i y_i. \tag{S42}$$

Because the second-order term in Eq. (S38) vanishes on linear functions, we obtain

$$\mathcal{L}_{\mathrm{FP}}(y_i \rho_{\mathrm{ss}}) = \mu_i \, y_i \rho_{\mathrm{ss}}. \tag{S43}$$

Thus $y_i(\boldsymbol{x})\rho_{\mathrm{ss}}(\boldsymbol{x})$ are eigenfunctions with eigenvalues μ_i.

Higher-order eigenvalues are obtained by examining the highest-degree part of a polynomial P. Let P_k be the homogeneous part of degree k of P. Since the linear functions y_i form a basis of linear polynomials, P_k is a linear combination of monomials

$$y_{i_1} y_{i_2} \cdots y_{i_k}, \tag{S44}$$

where i_j $(j \in \{1, \ldots, k\})$ is the integer corresponding to the eigenvector $\boldsymbol{v}_i$. For such a monomial, the first-order term in Eq. (S38) gives

$$(\tilde{A}\boldsymbol{x}) \cdot \nabla \left(y_{i_1} \cdots y_{i_k} \right) = \left(\mu_{i_1} + \cdots + \mu_{i_k} \right) y_{i_1} \cdots y_{i_k}, \tag{S45}$$

whereas the second-order term lowers the degree by two. Therefore

$$\mathcal{L}_{\mathrm{FP}} \left(y_{i_1} \cdots y_{i_k} \rho_{\mathrm{ss}} \right) = \left(\mu_{i_1} + \cdots + \mu_{i_k} \right) y_{i_1} \cdots y_{i_k} \rho_{\mathrm{ss}} + \rho_{\mathrm{ss}} R(\boldsymbol{x}), \tag{S46}$$

where $R(\boldsymbol{x})$ is a polynomial of degree at most $k-2$.

Finally, the lower-degree terms can be removed recursively. Specifically, one can choose

$$\widetilde{P} = y_{i_1} \cdots y_{i_k} + P_{k-2} + P_{k-4} + \cdots, \tag{S47}$$

with each P_m homogeneous of degree m, so that the lower-degree remainder cancels. Because the degree decreases by two at each step, this procedure terminates after finitely many steps. Hence $\widetilde{P}(\boldsymbol{x})\rho_{\mathrm{ss}}(\boldsymbol{x})$ satisfies

$$\mathcal{L}_{\mathrm{FP}}(\widetilde{P}\rho_{\mathrm{ss}}) = \left(\mu_{i_1} + \cdots + \mu_{i_k} \right) \widetilde{P}\rho_{\mathrm{ss}}. \tag{S48}$$

Therefore the eigenvalues of the Fokker–Planck operator are given by finite sums of the eigenvalues of A,

$$\lambda = \mu_{i_1} + \mu_{i_2} + \cdots + \mu_{i_k}. \tag{S49}$$

In particular, the oscillatory frequencies associated with the Fokker–Planck spectrum are determined by the imaginary parts of the eigenvalues of the deterministic drift matrix A.

Application of our decomposition to non-steady state dynamics

We demonstrate that our decomposition can be applied to non-steady-state dynamics. Specifically, we consider a relaxation process from an initial Gaussian distribution centered at the origin to the stationary distribution of the system. Figure S2a shows the time evolution of the probability density $p_t(\boldsymbol{x})$ governed by the Fokker–Planck equation (Eq. 2) at representative times. When the initial distribution is chosen as a Gaussian centered at the origin, the density progressively deforms and approaches the stationary distribution as time increases. Details of the numerical procedures and parameter settings used in this section are provided in Section *"Supplementary Methods"*.

To understand how the housekeeping entropy production rate σ_t^{hk} is generated during the relaxation process, we construct at each time the virtual dynamics driven by the instantaneous housekeeping local mean velocity field $\boldsymbol{\nu}_t^{\mathrm{hk}}$ as in Eq. 6. Examples of trajectories generated by these virtual dynamics are shown in Fig. S2b. Although the probability distribution $p_t(\boldsymbol{x})$ evolves in time and the system is therefore nonstationary, extracting the housekeeping component allows us to isolate the instantaneous sustaining directions of motion associated with the housekeeping entropy production rate. In this way, even in a time-dependent setting, the virtual dynamics reveal the oscillatory structure responsible for the housekeeping contribution at each moment. Notably, around $t \sim 10^{0.5}$, the probability distribution undergoes a pronounced change, and the qualitative structure of the virtual dynamics changes accordingly.

Figure S2c shows the time-dependent behavior of the total entropy production rate and its decomposition. The stacked bar plot represents the contributions from oscillatory modes $\sigma_t^{\mathrm{hk},(k)}$, while the white portion corresponds to the excess entropy production rate σ_t^{ex}. The gray line indicates the sum of the oscillatory contributions, and the black dashed line shows the housekeeping entropy production rate. Consistent with the structural change observed around $t = 10^{0.5}$, the excess entropy production rate increases during this transient stage. As the system approaches the stationary distribution, the excess contribution gradually diminishes and eventually vanishes, leaving only the housekeeping component for sufficiently large t, where the distribution is close to stationarity.

The frequency-resolved contributions to the housekeeping entropy production rate $\sigma_t^{\mathrm{hk},(k)}$ at representative times are shown in Fig. S2d. At early times, the contribution is dominated by a relatively limited set of frequencies. Around $t \sim 10^{0.5}$, the pattern of contributions changes qualitatively. For larger t, multiple oscillatory modes over a broader range of frequencies contribute to the housekeeping entropy production rate. As the distribution further approaches stationarity, the frequency structure gradually stabilizes toward that characteristic of the steady-state dynamics.

These results demonstrate that our decomposition consistently captures the evolving oscillatory structure of entropy production even in nonstationary settings.

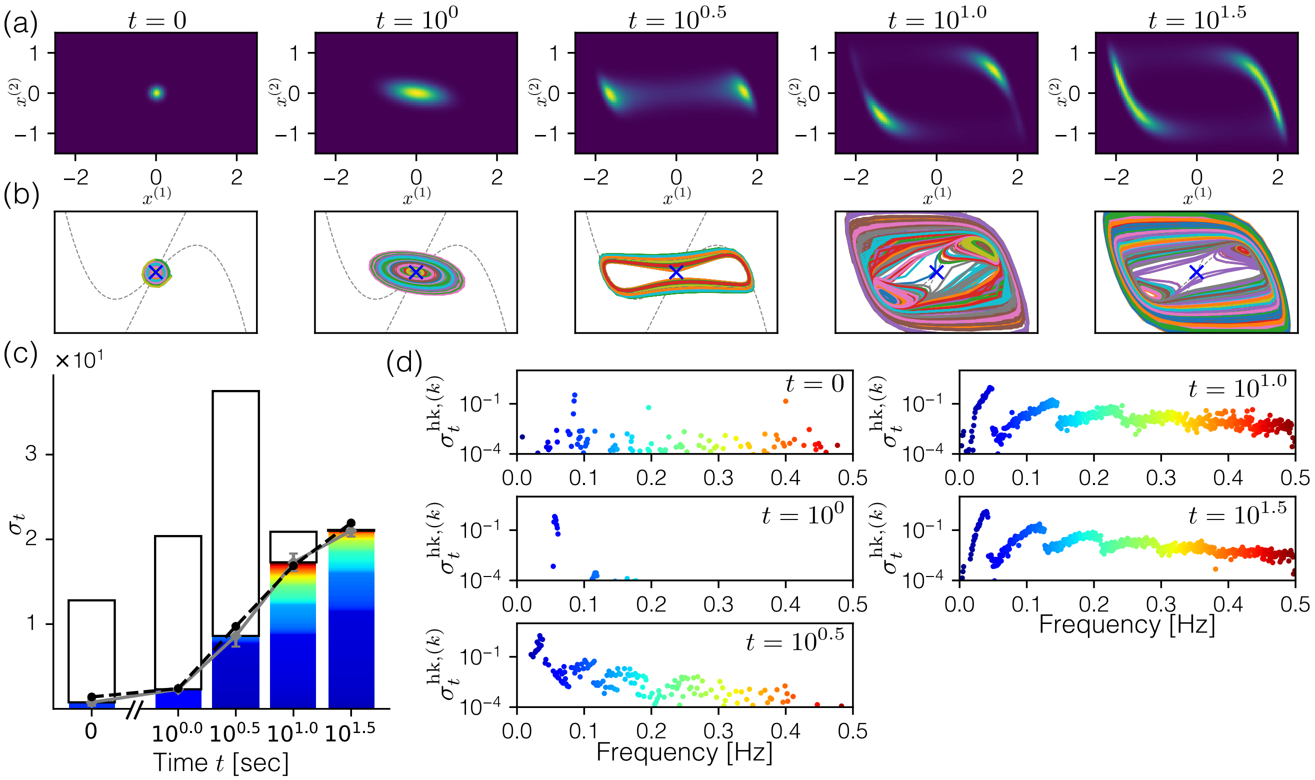

Fig. S2. Our decomposition enables us to analyze the time-dependent entropy production rate during a relaxation process from an initial Gaussian distribution centered at the origin to the stationary distribution. (a) Time evolution of the probability density $p_t(\boldsymbol{x})$ at representative times $t = 0,\ 10^0,\ 10^{0.5},\ 10^{1.0},\ 10^{1.5}$. (b) Examples of trajectories of the virtual dynamics in Eq. S1 constructed from the instantaneous housekeeping local mean velocity field $\boldsymbol{\nu}_t^{\mathrm{hk}}$ at each time. The dashed curves indicate the nullclines of the deterministic drift. The blue cross denotes the fixed point of the deterministic drift. (c) Time-dependent behavior of the entropy production rate and its decomposition. The stacked bar plot shows the contributions from oscillatory modes $\sigma_t^{\mathrm{hk},(k)}$, where the colors represent frequencies χ_k. The gray line indicates the sum of the contributions from the oscillatory modes, with error bars representing 95% confidence intervals. The black dashed line shows the true housekeeping entropy production rate. The white portion represents the excess entropy production rate σ_t^{ex}. (d) The contribution of each oscillatory mode to the housekeeping entropy production rate $\sigma_t^{\mathrm{hk},(k)}$ at different times t. Each panel corresponds to a representative time shown in (a).

Finite sampling effects in low-noise metastable systems

This section discusses how finite sampling affects the evaluation of our decomposition in low-noise metastable systems. The main point is that, in the numerical setting considered in the present study, this issue does not constitute a fundamental limitation because the true underlying Langevin dynamics are assumed to be known. Once the dynamics are specified, the stationary distribution and the local mean velocity field can be obtained directly from the discretized transition rate matrix on the state space grid, and additional trajectories can then be generated as needed. Therefore, insufficient sampling can in principle be remedied by increasing the number of sampled trajectories and checking convergence with respect to sample size.

At the same time, low-noise metastable systems still require particular care when the decomposition is evaluated from a finite number N of sampled trajectories. If N is too small, the sampled trajectories may not represent the full stationary dynamics sufficiently well, and the reconstructed entropy production can then be underestimated. To illustrate this point, we consider a low-noise bistable FitzHugh–Nagumo system with parameters $a = 0.1, b = 2.5, \tau = 12.5, R = 1, T = 10^{-1}, I = 0$.

Figure S3a shows example trajectories of the virtual dynamics for this parameter regime. The trajectories consist only of small motions around the two stable fixed points. As a result, when the number of sampled trajectories is too small, the sample can easily become biased toward one basin. This sampling bias affects the evaluation of the decomposition, even though the underlying stationary distribution and local mean velocity field themselves are known exactly in the present numerical framework.

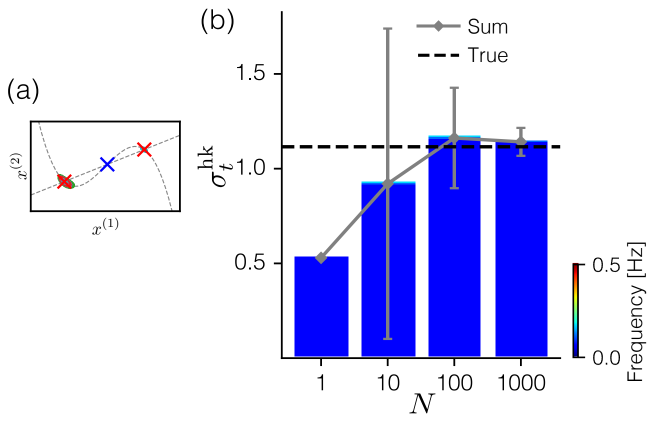

Fig. S3. (a) Examples of trajectories of the virtual dynamics in Eq. 6 for the noisy FitzHugh–Nagumo model in Eq. 13. The black dashed line represents the nullclines of the noisy FitzHugh–Nagumo model, which were calculated from the original Langevin dynamics in Eq. 13 by ignoring the noise term. The blue and red crosses represent the unstable and stable fixed points, respectively. (b) The N-dependent behavior of the housekeeping entropy production rate and its decomposition, where N represents the number of trajectories used to approximate the expectation in the calculation of our decomposition. The stacked bar plot shows the sum of the contributions from the oscillatory modes. The colors represent the frequencies of the oscillatory modes. The gray line indicates the sum of the contributions from the oscillatory modes, with error bars representing 95% confidence intervals. The black dashed line shows the true housekeeping entropy production rate.

Figure S3b shows how the housekeeping entropy production reconstructed from the decomposition depends on the number of sampled trajectories N. When N is small, the sum of the decomposed contributions underestimates the true housekeeping entropy production rate. As N is increased, the reconstructed value approaches the true value. At the same time, the confidence intervals become progressively narrower, indicating improved statistical reliability. For $N = 1$, the confidence interval is not shown because it is not defined.

These results show that finite sampling in low-noise metastable systems can cause underestimation of the reconstructed entropy production when too few trajectories are used. However, in the present numerical setting this is not a fundamental obstacle, because the true dynamics are known and additional trajectories can be generated as needed. Accordingly, the discrepancy can be reduced in practice by taking N sufficiently large and checking convergence with respect to sample size.

Supplementary Methods

This section describes the numerical procedures used for the noisy FitzHugh–Nagumo model (Sec. *"Applications to the noisy FitzHugh–Nagumo model"*). The numerical calculations consist of the following steps: (i) computing the housekeeping part of the local mean velocity $\boldsymbol{\nu}_t^{\mathrm{hk}}$ in the steady state, (ii) simulating the virtual dynamics driven by $\boldsymbol{\nu}_t^{\mathrm{hk}}$ in Eq. 6, and (iii) extracting Koopman eigenfunctions and modes from the generated time-series data and calculating the terms of our decomposition in Eq. S13. The numerical experiments were conducted under the following parameter settings for the noisy FitzHugh–Nagumo model. For Fig. 3a–e, we used $a = 0$, $b = 0.5$, $I = 0$, $T = 10^{-3}$, and $\tau = 12.5$. For Fig. 3f–h, the same parameters were used except that τ was varied from 2.5 to 22.5 in increments of 5. For Fig. 4, we set $a = 0$, $b = 0.5$, $T = 10^{-3}$, and $\tau = 12.5$, while varying I from 0 to 2.5 in increments of 0.1. For Fig. 6, we fixed $a = 0$, $b = 2$, $I = 0$, and $\tau = 12.5$, with T varied from 10^{-4} to 10^{-1} in logarithmic steps of 0.1.

Calculation of the local mean velocity. We calculated the housekeeping part of the local mean velocity $\boldsymbol{\nu}_t^{\mathrm{hk}}$ from the noisy FitzHugh–Nagumo model in Eq. 13. The decomposition was performed in the steady state, where the excess part vanishes, and the local mean velocity $\boldsymbol{\nu}_t$ coincides with its housekeeping part $\boldsymbol{\nu}_t^{\mathrm{hk}}$. Thus, determining the local mean velocity $\boldsymbol{\nu}_t$ is sufficient to obtain its housekeeping part $\boldsymbol{\nu}_t^{\mathrm{hk}}$. To compute the local mean velocity $\boldsymbol{\nu}_t$ at the steady state, we estimated the steady-state distribution p_t, which satisfies $\frac{\partial}{\partial t} p_t(\boldsymbol{x}) = 0$, using a discretization approach following the method in Ref. (6): the continuous Langevin equation in Eq. 13 was converted into a discrete transition rate matrix by dividing the state space into a grid. To discretize the state space, we divided the two variables of the FitzHugh–Nagumo model, $x^{(1)}$ and $x^{(2)}$, into a grid. The variable $x^{(1)}$ was discretized into 10^4 intervals ranging from -5 to 5, and $x^{(2)}$ was discretized into 10^4 intervals spanning $-5+I$ to $5+I$. This resulted in a total of $10^{4\times 2}$ grid points. In this discrete system with $10^{4\times 2}$ states, we constructed the transition rate matrix corresponding to the Langevin equation in Eq. 13. The steady-state distribution for the grid points was then obtained by computing the eigenvector corresponding to the zero eigenvalue of the transition rate matrix. From this steady-state distribution, we computed the gradient $\nabla \ln p_t$ by interpolating p_t between grid points using cubic-spline interpolation and differentiating the resulting cubic polynomial. This method allowed us to compute the local mean velocity not only at the grid points but also at arbitrary locations within the continuous state space.

Simulating the virtual dynamics. Having obtained the housekeeping part of the local mean velocity $\boldsymbol{\nu}_t^{\mathrm{hk}}$, we generated time-series trajectories by simulating the virtual dynamics driven by $\boldsymbol{\nu}_t^{\mathrm{hk}}$ in Eq. (6). We simulated the dynamics using an eighth-order Runge–Kutta method with a time step $\Delta s = 1$, generating trajectories of length $S = 150$ steps. For each parameter setting of the noisy FitzHugh–Nagumo model, we generated $N = 1000$ independent trajectories to evaluate the decomposition of the entropy production rate. These trajectories serve as Monte Carlo samples for approximating the terms in our decomposition. The initial conditions for the trajectories were sampled from the discretized steady-state distribution $p_t(\boldsymbol{x})$. Hereafter, we denote by $\boldsymbol{x}_{n,s}$ the state at time s of the n-th trajectory.

Extraction of Koopman eigenfunctions and modes. From the simulated time-series data, we estimated the Koopman eigenfunctions $\{\phi_{n,k}\}_{k=1}^{r_n}$, eigenvalues $\{\lambda_{n,k}\}_{k=1}^{r_n}$, and modes $\{\boldsymbol{v}_{n,k}\}_{k=1}^{r_n}$ for each trajectory. Because different trajectories have different supports in state space, the eigenfunctions obtained from distinct trajectories were regarded as different functions. Here, r_n denotes the number of extracted modes for the n-th trajectory, and the double subscript $_{n,k}$ indicates the k-th eigenfunction, eigenvalue, or mode associated with that trajectory. The number of extracted modes r_n varies across trajectories, and the procedure for determining r_n is described later. To obtain these quantities, we employed Hankel DMD (7–9) in combination with physics-informed DMD (PiDMD) (10) via the PyDMD Python package (11). Hankel DMD constructs a vector of h_n observable functions, $\boldsymbol{g}(\boldsymbol{x}) = (\boldsymbol{g}_1(\boldsymbol{x})^\top, \boldsymbol{g}_2(\boldsymbol{x})^\top, \ldots, \boldsymbol{g}_{h_n}(\boldsymbol{x})^\top)^\top = (\mathrm{Id}(\boldsymbol{x})^\top, (e^{\Delta s \mathcal{K}} \mathrm{Id}(\boldsymbol{x}))^\top, \ldots, (e^{(h_n-1)\Delta s \mathcal{K}} \mathrm{Id}(\boldsymbol{x}))^\top)^\top \in \mathbb{R}^{dh_n}$, where $\mathrm{Id}(\boldsymbol{x}) = \boldsymbol{x}$ is the identity function. Here, h_n denotes the number of time delays chosen for the n-th trajectory, which serves as a hyperparameter of the fitting procedure. The procedure for selecting h_n is also described later. This vector is obtained by a time-delay embedding of the time-series data, i.e., $\boldsymbol{g}(\boldsymbol{x}_{n,s}) = (\boldsymbol{x}_{n,s}^\top, \boldsymbol{x}_{n,s+\Delta s}^\top, \ldots, \boldsymbol{x}_{n,s+(h_n-1)\Delta s}^\top)^\top$. Since the Koopman generator $\mathcal{K}$ is linear, the time evolution of this observable vector can be approximated by a linear dynamical system, even when the dynamics of $\boldsymbol{x}_{n,s}$ are nonlinear.

To estimate the Koopman generator $\mathcal{K}$ while ensuring that its eigenvalues are purely imaginary, we applied PiDMD, which constrains the representation matrix of $e^{\Delta s \mathcal{K}}$ to be unitary during the fitting procedure. Let L denote the representation matrix of $e^{\Delta s \mathcal{K}}$. Under stationarity, the covariance matrix Σ of the observable vectors satisfies $\Sigma = L \Sigma L^*$, which implies that when Σ is the identity matrix, L must be unitary. Therefore, before applying PiDMD, we linearly transformed the delay-embedded data so that its covariance matrix became the identity matrix, ensuring that the fitted L satisfies the unitarity condition under stationarity. Specifically, we first centered the delay-embedded observable vectors by subtracting their temporal mean, $\overline{\boldsymbol{g}} = (1/(S-h_n+1)) \sum_{s=1}^{S-h_n+1} \boldsymbol{g}(\boldsymbol{x}_{n,s})$, $G = (\boldsymbol{g}(\boldsymbol{x}_{n,1}) - \overline{\boldsymbol{g}},\ \boldsymbol{g}(\boldsymbol{x}_{n,2}) - \overline{\boldsymbol{g}},\ \ldots,\ \boldsymbol{g}(\boldsymbol{x}_{n,S-h_n+1}) - \overline{\boldsymbol{g}})$ and then performed a singular value decomposition $G = USV^\top$. We whitened the data as $\tilde{G} = S^{-1} U^\top G$, and applied PiDMD to the new data matrix $\tilde{G}$ to obtain the Koopman modes $\{\tilde{\boldsymbol{v}}_{n,k}\}$. Here, the number of extracted modes r_n was determined for each trajectory from this singular value decomposition by applying optimal singular value hard thresholding (SVHT) (12) to the singular values of G. Finally, the obtained modes were mapped back to the original coordinate system as $\boldsymbol{v}_{n,k} = US\tilde{\boldsymbol{v}}_{n,k}$, and the temporal mean $\overline{\boldsymbol{g}}$ was added back to the reconstructed trajectories so that they could be interpreted in the original observable space $\boldsymbol{g}(\boldsymbol{x}_{n,s})$.

For each trajectory, the number of time delays h_n was chosen from the range of 1 to 100 in increments of 1 to minimize the reconstruction error based on Eq. S9, $\sum_{s=1}^{S} \left\| \boldsymbol{x}_{n,s} - \sum_{k=1}^{r_n} e^{\lambda_{n,k} s} \phi_{n,k}(\boldsymbol{x}_{n,0})\, \boldsymbol{v}_{n,k} \right\|^2$, which varies with the delay dimension h_n because the extracted eigenvalues $\lambda_{n,k}$, eigenfunctions $\phi_{n,k}$, and modes $\boldsymbol{v}_{n,k}$ depend on the chosen h_n during the fitting procedure.

Computation of the terms of our decomposition. From the Koopman eigenvalues, eigenfunctions, and modes obtained for each trajectory, we computed the terms of our decomposition as follows:

$$\chi_{n,k} = |\lambda_{n,k}/(2\pi \mathrm{i})| \qquad \text{[S50]}$$

$$J_{n,k} = \frac{1}{S} \sum_{s} (\phi_{n,k}(\boldsymbol{x}_{n,s}) \boldsymbol{v}_{n,k})^* D_t^{-1} (\phi_{n,k}(\boldsymbol{x}_{n,s}) \boldsymbol{v}_{n,k}), \qquad \text{[S51]}$$

$$\sigma_t^{\mathrm{hk},(n,k)} = (2\pi)^2 \chi_{n,k}^2 J_{n,k}, \qquad \text{[S52]}$$

where the expected values in Eq. S13 are approximated as the time-average within the trajectory. Since each eigenfunction is supported only within its corresponding trajectory and does not overlap with those from other trajectories, the associated quantities $\chi_{n,k}, J_{n,k}$, and $\sigma_t^{\mathrm{hk},(n,k)}$ were considered as distinct terms for each trajectory. Let $r = \sum_{n=1}^{N} r_n$ denote the total number of extracted modes across all trajectories. To summarize the results in a form consistent with ensemble expectations, we concatenated these quantities and reindexed them, dividing each trajectory-specific contribution by N: $\{J_{1,1}/N, ..., J_{1,r_1}/N, ..., J_{N,1}/N, ..., J_{N,r_N}/N\}$, $\{\sigma_t^{\mathrm{hk},(1,1)}/N, ..., \sigma_t^{\mathrm{hk},(1,r_1)}/N, ..., \sigma_t^{\mathrm{hk},(N,1)}/N, ..., \sigma_t^{\mathrm{hk},(N,r_N)}/N\}$. These reindexed quantities were then plotted in Figs. 3, 4, and 6. In the frequency-resolved panels in Figs. 3d, g, h; 4c; 6d, we applied a moving-window procedure to enable clearer comparison across frequencies: this both alleviates overlap among points and compensates for non-uniform point densities across frequency bands. Within each bin of width 10^{-3}, the values were summed and plotted at the bin center.

Estimation of confidence intervals. To assess the finite-sample variability of the sum of the decomposition, we treated $\{\sum_{k=1}^{r_n} \sigma_t^{\mathrm{hk},(n,k)}\}_{n=1}^{N}$ as N independent samples. For clarity, we define the trajectory-wise sum of the decomposition as $\sigma_n^{\mathrm{sum}} = \sum_{k=1}^{r_n} \sigma_t^{\mathrm{hk},(n,k)}$. The sample mean $\bar{\sigma}^{\mathrm{sum}} = \frac{1}{N}\sum_{n=1}^{N} \sigma_n^{\mathrm{sum}}$ and the standard error $\mathrm{SE}^{\mathrm{sum}} = \sqrt{\frac{1}{N(N-1)}\sum_{n=1}^{N}(\sigma_n^{\mathrm{sum}} - \bar{\sigma}^{\mathrm{sum}})^2}$ were then computed. From these, 95% confidence intervals were constructed as

$$\bar{\sigma}^{\mathrm{sum}} \pm t_{N-1,\,0.975}\,\mathrm{SE}^{\mathrm{sum}}, \tag{S53}$$

where $t_{N-1,0.975}$ denotes the 97.5% quantile of the t-distribution with $N-1$ degrees of freedom. These confidence intervals are shown as error bars for the sum of the decomposition in Figs. 3–6.

Calculation of the true values of the housekeeping entropy production rates. To validate that the sum of our decomposition recovers the true value, we computed the true housekeeping entropy production rate using the method of Ref. (6). Specifically, with the steady-state distribution $p_t(\boldsymbol{x})$ and the local mean velocity field $\boldsymbol{\nu}_t^{\mathrm{hk}}(\boldsymbol{x})$ obtained as described above, the true housekeeping entropy production rate was calculated, following the definition in Eq. 5, by numerically integrating $\boldsymbol{\nu}_t^{\mathrm{hk}}(\boldsymbol{x})^\top D_t^{-1} \boldsymbol{\nu}_t^{\mathrm{hk}}(\boldsymbol{x})\, p_t(\boldsymbol{x})$. This result is shown in Fig. 3e and as the black dashed lines in Figs. 3f, 4b, and 6c.

Calculation of correlation times. The correlation time τ_{corr} in Fig. 6b represents the degree of temporal coherence in dynamical systems, and serves as a signature of coherent resonance (13). It is theoretically defined as

$$\tau_{\mathrm{corr}} = \int_0^\infty C(u)^2\, du, \tag{S54}$$

where $C(u) := \mathrm{Cov}[x_{t'}^{(1)}, x_{t'+u}^{(1)}]/\mathrm{Var}[x_{t'}^{(1)}]$ represents the autocorrelation function of $x_t^{(1)}$ with the time lag of u. Here, $\mathrm{Cov}[x_{t'}^{(1)}, x_{t'+u}^{(1)}]$ is the covariance between $x_{t'}^{(1)}$ and $x_{t'+u}^{(1)}$ in the steady state, and $\mathrm{Var}[x^{(1)}]$ is the variance of $x^{(1)}$ in the steady state. Because the process is in the steady state, these quantities do not depend on the choice of the reference time t', but only on the lag u. Since τ_{corr} integrates the squared autocorrelation over time, a higher value indicates that correlations decay more slowly, corresponding to more temporally ordered dynamics.

The correlation times were numerically calculated as follows. We simulated $N_{\mathrm{sr}} = 1000$ independent trajectories $\{\boldsymbol{x}_{n,t}\}_{t=1}^{S_{\mathrm{sr}}}$ of the Langevin dynamics in Eq. 13 by the Euler–Maruyama method, with a step size $\Delta t = 10^{-2}$ and $S_{\mathrm{sr}} = 10^4$ steps for each trajectory n. The initial conditions of these simulations were sampled from the stationary distribution of Eq. 13, which was also used in the numerical calculation of the decomposition. Note that these trajectories differ from those following the virtual deterministic process in Eq. 6 used in the decomposition analysis. From the first component $x_{n,t}^{(1)}$ of each trajectory, we computed the autocorrelation function at lag $\ell\Delta t$ $(0 \le \ell \le S_{\mathrm{sr}} - 1,\ \ell \in \mathbb{Z}_{\ge 0})$ as

$$C_n(\ell\Delta t) = \frac{\frac{1}{S_{\mathrm{sr}}-\ell}\sum_{j=1}^{S_{\mathrm{sr}}-\ell}\left(x_{n,j\Delta t}^{(1)} - \bar{x}_n^{(1)}\right)\left(x_{n,(j+\ell)\Delta t}^{(1)} - \bar{x}_n^{(1)}\right)}{\frac{1}{S_{\mathrm{sr}}}\sum_{j=1}^{S_{\mathrm{sr}}}\left(x_{n,j\Delta t}^{(1)} - \bar{x}_n^{(1)}\right)^2}, \tag{S55}$$

where $\bar{x}_n^{(1)} = \frac{1}{S_{\mathrm{sr}}}\sum_{j=1}^{S_{\mathrm{sr}}} x_{n,j\Delta t}^{(1)}$ is the time average within trajectory n. This estimator of the autocorrelation is motivated by the fact that, in the steady state, the variance does not depend on the time index, and the covariance depends only on the lag $\ell\Delta t$ but not on the reference time $j\Delta t$. The trajectory-specific correlation time was then obtained by summing the squared autocorrelation over lags,

$$\tau_{\mathrm{corr},n} = \sum_{\ell=0}^{S_{\mathrm{sr}}-1} C_n(\ell\Delta t)^2\,\Delta t. \tag{S56}$$

The mean correlation time across trajectories was calculated as $\tau_{\mathrm{corr}} = N_{\mathrm{sr}}^{-1}\sum_{n=1}^{N_{\mathrm{sr}}} \tau_{\mathrm{corr},n}$ and plotted in Fig. 6b, with error bars indicating the 95% confidence interval. The confidence intervals were constructed by evaluating the variability among $\{\tau_{\mathrm{corr},n}\}_{n=1}^{N_{\mathrm{sr}}}$ using the t-distribution with $N_{\mathrm{sr}} - 1$ degrees of freedom.

Comparison with the limit-cycle frequency. For the results shown in Fig. S1b-d, we estimated the oscillation frequency directly from simulations of the noisy FitzHugh–Nagumo model in Eq. 13. The parameter values were set to $a = 0$, $b = 0.5$, $I = 0$, $T = 10^{-4}$, and $\tau = 12.5$. Under this parameter setting, the deterministic part of the dynamics exhibits limit-cycle oscillations, and the stochastic trajectories fluctuate around this cycle.

To estimate the oscillation frequency, we simulated $N_{\mathrm{lc}} = 1000$ independent trajectories of the noisy FitzHugh–Nagumo dynamics using the Euler–Maruyama method with time step $\Delta t = 10^{-2}$ for $S_{\mathrm{lc}} = 10^5$ steps. We denote the state of the n-th trajectory at step s by $\boldsymbol{x}_{n,s} = (x_{n,s}^{(1)}, x_{n,s}^{(2)})$.

For each trajectory, we introduced a phase variable defined from the two-dimensional state,

$$\theta_{n,s} = \arg\left((x_{n,s}^{(1)} - \bar{x}_n^{(1)}) + i(x_{n,s}^{(2)} - \bar{x}_n^{(2)})\right), \qquad \text{[S57]}$$

where $\bar{x}_n^{(j)} = \frac{1}{S_{\mathrm{lc}}} \sum_{s=1}^{S_{\mathrm{lc}}} x_{n,s}^{(j)}$ denotes the temporal mean of the j-th component along trajectory n. The phase sequence $\{\theta_{n,s}\}$ was unwrapped to remove discontinuities at multiples of 2π.

The oscillation frequency for trajectory n was then estimated from the long-time average rate of phase increase. Specifically, we computed the average amount by which the phase advances per unit time along the trajectory,

$$\omega_n = \frac{\theta_{n,S_{\mathrm{lc}}} - \theta_{n,1}}{(S_{\mathrm{lc}} - 1)\Delta t}, \qquad \text{[S58]}$$

which represents the mean angular velocity of the trajectory. The corresponding oscillation frequency was then obtained as

$$f_n = \frac{\omega_n}{2\pi}. \qquad \text{[S59]}$$

Finally, the limit-cycle frequency used for comparison with the dominant oscillatory mode of the housekeeping entropy production rate was obtained by averaging over trajectories,

$$f_{\mathrm{LC}} = \frac{1}{N_{\mathrm{lc}}} \sum_{n=1}^{N_{\mathrm{lc}}} f_n. \qquad \text{[S60]}$$

Comparison with the frequencies extracted by linear stability analysis. For the comparison with the frequencies predicted by linear stability analysis in Fig. 5 and Fig. S1, we computed the frequency determined by the fixed-point analysis of the virtual dynamics driven by the housekeeping local mean velocity field $\boldsymbol{\nu}_t^{\mathrm{hk}}$.

Using the velocity field $\boldsymbol{\nu}_t^{\mathrm{hk}}$ obtained from the discretized state space and its interpolation as described above, the fixed point $\boldsymbol{x}_{\mathrm{fix}}$ of the virtual dynamics was obtained by minimizing $\|\boldsymbol{\nu}_t^{\mathrm{hk}}(\boldsymbol{x})\|^2$, which identifies the point where $\boldsymbol{\nu}_t^{\mathrm{hk}}(\boldsymbol{x}_{\mathrm{fix}}) = \mathbf{0}$. After locating the fixed point, the Jacobian $\partial \boldsymbol{\nu}_t^{\mathrm{hk}} / \partial \boldsymbol{x}\big|_{\boldsymbol{x}=\boldsymbol{x}_{\mathrm{fix}}}$ was evaluated numerically using a central finite-difference approximation with step size $h = 10^{-6}$.

The frequency predicted by linear stability analysis was then obtained from

$$f_{\mathrm{LSA}} = \frac{1}{2\pi} \left|\mathrm{Im}(\lambda_{\mathrm{LSA}})\right|, \qquad \text{[S61]}$$

where λ_{LSA} is an eigenvalue of the Jacobian $\partial \boldsymbol{\nu}_t^{\mathrm{hk}} / \partial \boldsymbol{x}\big|_{\boldsymbol{x}=\boldsymbol{x}_{\mathrm{fix}}}$. In the present two-dimensional system, if the eigenvalues have nonzero imaginary parts, they form a complex-conjugate pair, so either eigenvalue gives the same absolute value of the imaginary part. This frequency was used for comparison with the dominant oscillatory mode extracted by our decomposition in Fig. 5 and Fig. S1.

Application of our decomposition to non-steady state dynamics. For the results shown in Fig. S2, we analyzed a non-steady-state relaxation process of the noisy FitzHugh–Nagumo model in Eq. 13 with parameters $a = 0$, $b = 0.5$, $I = 0$, $T = 10^{-3}$, and $\tau = 12.5$. The initial distribution was chosen as a Gaussian distribution $p_0(\boldsymbol{x}) = \mathcal{N}\left(\begin{pmatrix} 0 \\ 0 \end{pmatrix}, \begin{pmatrix} 0.1 & 0 \\ 0 & 0.1 \end{pmatrix}\right)$. The time evolution of the probability distribution was first computed on a discretized state space. At representative time points during the relaxation process (Fig. S2a), we evaluated the instantaneous velocity field and decomposed it into excess and housekeeping components. Using the resulting housekeeping velocity field $\boldsymbol{\nu}_t^{\mathrm{hk}}$, we constructed the corresponding virtual dynamics and generated $N = 1000$ trajectories of length $S = 150$ steps from initial conditions sampled from the instantaneous distribution $p_t(\boldsymbol{x})$. The Koopman-mode decomposition was then applied to these trajectories to compute the frequency-resolved contributions to the housekeeping entropy production rate in Fig. S2c-d.

To compute the time evolution of the probability distribution, the state space was discretized on a uniform grid over $[-5, 5] \times [-5, 5]$ with 2000×2000 grid points, and the corresponding transition-rate matrix W was constructed in the same manner as in the steady-state analysis. Let $\boldsymbol{P}_t$ denote the vector obtained by flattening the discretized probability distribution on the grid. Starting from the initial vector $\boldsymbol{P}_0$, the probability distribution was evolved according to

$$\frac{\partial \boldsymbol{P}_t}{\partial t} = W \boldsymbol{P}_t, \qquad \text{[S62]}$$

which corresponds to the discretized Fokker–Planck equation. This equation was solved using the stiff ODE solver `ode15s`, and the probability distribution was evaluated at the representative times shown in Fig. S2a.

For each time point, the instantaneous velocity field was decomposed into excess and housekeeping components. To obtain the excess component, we introduced a scalar potential $\phi(\boldsymbol{x})$ and defined the excess velocity field as the gradient flow $\boldsymbol{\nu}_t^{\mathrm{ex}} = \nabla\phi$. The potential was determined so that the continuity equation

$$\frac{\partial p_t(\boldsymbol{x})}{\partial t} = -\nabla \cdot (p_t(\boldsymbol{x})\nabla\phi(\boldsymbol{x})) \qquad \text{[S63]}$$

is satisfied, where $\boldsymbol{x} = (x^{(1)}, x^{(2)})$. On the discretized grid, let $\boldsymbol{\phi}$ denote the vector obtained by evaluating $\phi(\boldsymbol{x})$ at the grid points. Introducing finite-difference operators G_1 and G_2 that approximate the spatial derivatives $\partial/\partial x^{(1)}$ and $\partial/\partial x^{(2)}$, the discretized form of the above continuity equation can be written as

$$\frac{\partial \boldsymbol{P}_t}{\partial t} = -\left(G_1^\top \mathrm{diag}(\boldsymbol{P}_t)G_1 + G_2^\top \mathrm{diag}(\boldsymbol{P}_t)G_2\right)\boldsymbol{\phi}. \qquad \text{[S64]}$$

Here $\mathrm{diag}(\boldsymbol{P}_t)$ denotes the diagonal matrix whose diagonal elements are given by the components of $\boldsymbol{P}_t$. This equation corresponds to the discretized form of the continuity equation above. We solved this linear equation for $\boldsymbol{\phi}$ using a least-squares procedure. The excess velocity field on the grid was then obtained from

$$\nu_t^{\mathrm{ex},(1)} = G_1\boldsymbol{\phi}, \qquad \nu_t^{\mathrm{ex},(2)} = G_2\boldsymbol{\phi}. \qquad \text{[S65]}$$

The excess entropy production rate shown in Fig. S2c was computed numerically on the grid using this excess velocity field together with the discretized probability distribution. The housekeeping component was then defined as the residual

$$\boldsymbol{\nu}_t^{\mathrm{hk}} = \boldsymbol{\nu}_t - \boldsymbol{\nu}_t^{\mathrm{ex}}. \qquad \text{[S66]}$$

At $t = 10^{0.5}$, where the probability distribution changes rapidly, the numerical accuracy of the velocity field becomes more sensitive to the grid resolution. For this time point only, after computing the distribution on the 2000×2000 grid, it was refined to a 3000×3000 grid by cubic-spline interpolation before constructing the virtual dynamics. After simulating $N = 1000$ trajectories from the resulting velocity field, we excluded trajectories that did not return sufficiently close to their initial points during the final part of the orbit. Specifically, for each trajectory we computed the minimum squared distance between the initial point and the last 30 time steps, and retained only those satisfying

$$\min_{s\in\{S-29,\ldots,S\}} \|\boldsymbol{x}_{n,s} - \boldsymbol{x}_{n,0}\|^2 < 10^{-4}. \qquad \text{[S67]}$$

For each time point, the Koopman eigenvalues, eigenfunctions, and modes were extracted from the virtual trajectories in the same manner as in the steady-state analysis. From these quantities, we computed the frequency-resolved contributions $\sigma_t^{\mathrm{hk},(n,k)}$ and aggregated them across trajectories. The quantities plotted in Fig. S2d correspond to these frequency-resolved contributions. The stacked bars in Fig. S2c were obtained by summing the housekeeping contributions over oscillatory modes and adding the excess entropy production rate.

References

1. BO Koopman, Hamiltonian systems and transformation in hilbert space. *Proc. Natl. Acad. Sci.* **17**, 315–318 (1931).
2. I Mezić, Analysis of fluid flows via spectral properties of the koopman operator. *Annu. review fluid mechanics* **45**, 357–378 (2013).
3. D Sekizawa, S Ito, M Oizumi, Decomposing thermodynamic dissipation of linear langevin systems via oscillatory modes and its application to neural dynamics. *Phys. Rev. X* **14**, 041003 (2024).
4. D Liberzon, RW Brockett, Spectral analysis of fokker–planck and related operators arising from linear stochastic differential equations. *SIAM J. on Control. Optim.* **38**, 1453–1467 (2000).
5. TK Leen, R Friel, D Nielsen, Eigenfunctions of the multidimensional linear noise fokker-planck operator via ladder operators. *arXiv preprint arXiv:1609.01194* (2016).
6. TR Gingrich, GM Rotskoff, JM Horowitz, Inferring dissipation from current fluctuations. *J. Phys. A: Math. Theor.* **50**, 184004 (2017).
7. JH Tu, CW Rowley, DM Luchtenburg, SL Brunton, JN Kutz, On dynamic mode decomposition: Theory and applications (2014).
8. H Arbabi, I Mezic, Ergodic theory, dynamic mode decomposition, and computation of spectral properties of the koopman operator. *SIAM J. on Appl. Dyn. Syst.* **16**, 2096–2126 (2017).
9. SL Brunton, BW Brunton, JL Proctor, E Kaiser, JN Kutz, Chaos as an intermittently forced linear system. *Nat. communications* **8**, 19 (2017).
10. PJ Baddoo, B Herrmann, BJ McKeon, J Nathan Kutz, SL Brunton, Physics-informed dynamic mode decomposition. *Proc. Royal Soc. A* **479**, 20220576 (2023).
11. SM Ichinaga, et al., Pydmd: A python package for robust dynamic mode decomposition. *J. Mach. Learn. Res.* **25**, 1–9 (2024).
12. M Gavish, DL Donoho, The optimal hard threshold for singular values is $4/\sqrt{3}$. *IEEE Transactions on Inf. Theory* **60**, 5040–5053 (2014).
13. AS Pikovsky, J Kurths, Coherence resonance in a noise-driven excitable system. *Phys. Rev. Lett.* **78**, 775 (1997).
